# Integrated Strengthening of Recycled Coarse Aggregates and Cementitious Matrix Optimization for Concrete and Cement-Stabilized Materials from Construction and Demolition Waste

**DOI:** 10.3390/ma19153238

**Published:** 2026-07-30

**Authors:** Lingtong Zhang, Zhen Zhang, Liming Zhang, Baoyuan Li, Dandan Shen, Chuangzhou Wu

**Affiliations:** 1 School of Smart Water Conservancy Engineering, Xinjiang Institute of Technology, Aksu 843100, China; zhqling2024@163.com (L.Z.); zz891010zz@163.com (Z.Z.); 15899229636@163.com (B.L.); 15829723653@163.com (D.S.); 2 Ocean College, Zhejiang University, Zhoushan 316021, China; ark_wu@zju.edu.cn

**Keywords:** recycled coarse aggregate, construction and demolition waste, aggregate modification, cement-stabilized materials, mechanical properties, durability

## Abstract

To promote the high-value utilization of construction and demolition waste in cementitious materials, this study proposed an integrated strengthening strategy combining recycled coarse aggregate modification with cementitious matrix optimization. Recycled coarse aggregates with particle sizes of 4.75–31.5 mm were prepared from demolished concrete waste collected in Aksu, Xinjiang, China, and treated by particle shaping, alkaline solutions, and polyvinyl alcohol (PVA). Silica fume and a polycarboxylate superplasticizer were used to optimize the cementitious matrix. The physical properties of recycled aggregates, the mechanical performance of recycled aggregate concrete, and the mechanical and durability performance of high-content recycled aggregate cement-stabilized materials were evaluated. Particle shaping reduced water absorption and the crushing index from 6.8% and 14.4% to 5.6% and 12.6%, respectively. After treatment with 5% NaOH and 10% PVA, the apparent density increased to 2779 kg/m^3^, whereas water absorption and the crushing index decreased to 3.3% and 8.3%, meeting Class II recycled coarse aggregate requirements. With 10% silica fume and 0.2% superplasticizer, the 28-day compressive strength of recycled aggregate concrete prepared with the optimized aggregate and matrix reached 27.6 MPa, corresponding to 97.5% of that of natural aggregate concrete. The combined modification approach improved mechanical performance and reduced drying shrinkage, but the replacement ratio should be limited to 60% to satisfy the F50 freeze–thaw requirement under the tested conditions.

## 1. Introduction

Rapid urbanization and infrastructure development have greatly increased the demand for natural aggregates and generated large quantities of construction and demolition waste. Excessive extraction of natural sand and gravel may lead to resource depletion, ecological disturbance, and rising construction costs. In contrast, the landfilling or open-air stockpiling of construction and demolition waste occupies land resources and poses potential environmental risks [1,2,3]. Therefore, converting construction and demolition waste into recycled aggregates for concrete production is considered an important strategy for reducing the consumption of natural aggregates and promoting the development of sustainable construction materials [4,5,6].

**Figure 1:**
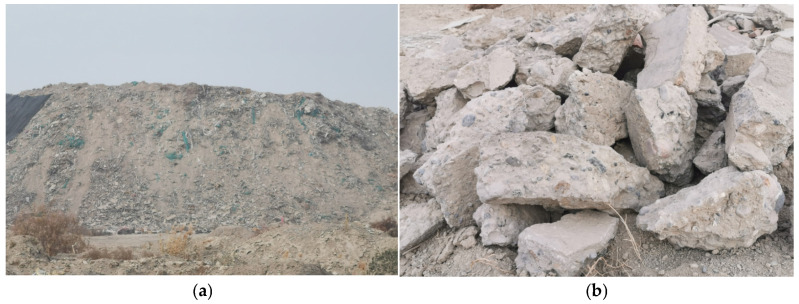
Field photographs of construction and demolition waste in Aksu City: (**a**) open-air stockpiling site in the suburban area of Aksu; (**b**) typical construction and demolition waste.

In rapidly developing inland regions, the recycling and high-value utilization of construction and demolition waste remain insufficient. Taking Aksu City in Xinjiang, China, as an example, urban renewal and infrastructure construction have produced considerable amounts of construction solid waste. In current practice, part of this waste is transported to suburban areas for open-air stockpiling or landfill disposal, resulting in land occupation, environmental pressure, and resource loss, as shown in Figure 1. Therefore, using local construction and demolition waste to produce recycled coarse aggregates is of practical significance for improving waste utilization efficiency and supporting low-carbon construction in this region.

Recycled coarse aggregates have been widely investigated as substitutes for natural aggregates in concrete. However, compared with natural aggregates, recycled aggregates generally exhibit higher water absorption, higher crushing index, lower density, and greater variability in quality [7,8,9,10]. These deficiencies are mainly caused by residual old mortar attached to the aggregate surface, microcracks generated during crushing, and the porous structure of the recycled aggregate–mortar system [11,12]. When recycled aggregates are incorporated into concrete, these characteristics may increase water demand, weaken the interfacial transition zone, and reduce the mechanical properties and durability of recycled aggregate concrete [13,14,15,16]. These adverse effects become more pronounced when the replacement ratio of recycled coarse aggregate is high [17,18].

To improve the quality of recycled aggregates, various physical treatment methods have been proposed. Mechanical grinding, rubbing, abrasion, and particle shaping can remove part of the loosely adhered mortar from the surface of recycled aggregates and improve particle morphology [19,20,21]. These methods are generally effective in reducing water absorption and crushing index and improving aggregate packing characteristics. However, physical treatment alone cannot completely remove residual mortar or repair internal microcracks. Excessive mechanical treatment may also damage aggregate particles or increase processing energy consumption. Therefore, physical treatment is often regarded as a preliminary strengthening method rather than a complete solution for high-quality recycled aggregate production.

Chemical treatment has been used to modify recycled aggregates through carbonation, silicate impregnation, inorganic slurry coating, and other surface treatments [22,23,24,25,26]. These approaches may improve aggregate quality by altering the attached mortar layer, reducing pore connectivity, or densifying the aggregate surface. However, their effectiveness depends strongly on the treatment agent, concentration, exposure duration, washing procedure, and drying conditions.

Polymer-based modification provides another route for improving recycled aggregate quality. Polymer impregnation or coating can reduce water absorption and improve surface integrity by partially sealing accessible pores and microcracks [27,28,29].

Previous studies have mainly examined mechanical treatment, chemical treatment, and polymer modification as separate or individually emphasized approaches. Alkaline treatment and PVA-based modification have each been reported for recycled aggregates, but the present study focuses on a defined sequential route involving particle shaping, alkaline immersion, washing, drying, and subsequent PVA modification. This route was further evaluated using recycled coarse aggregates in both recycled aggregate concrete and high-content cement-stabilized materials.

In addition to aggregate modification, cementitious matrix optimization can improve the performance of recycled aggregate-based materials. Silica fume can refine the pore structure and improve the interfacial transition zone through micro-filling and pozzolanic effects [30], while polycarboxylate-based water-reducing agents can improve workability without increasing the mixing-water demand. However, the relationship among aggregate strengthening, matrix densification, mechanical performance, freeze–thaw resistance, and drying shrinkage remains insufficiently clarified, particularly for materials prepared with high recycled aggregate contents [31,32,33,34].

Based on these considerations, this study proposes an integrated strengthening strategy that links recycled coarse aggregate modification with cementitious matrix optimization. Recycled coarse aggregates prepared from construction and demolition waste in Aksu City were subjected to particle shaping, alkaline treatment, and PVA modification, while silica fume and a polycarboxylate-based water-reducing agent were incorporated to optimize the cementitious matrix. The objectives were to: (1) evaluate the effects of different strengthening treatments on the physical properties of recycled coarse aggregates; (2) compare NaOH and Na_2_CO_3_ treatments and identify suitable treatment parameters; (3) assess the mechanical properties and microstructural characteristics of recycled aggregate concrete, as well as the mechanical properties, freeze–thaw resistance, and drying shrinkage behavior of high-content recycled aggregate cement-stabilized materials; and (4) discuss the possible improvement mechanisms and determine a reasonable recycled coarse aggregate replacement ratio for engineering applications in severely cold regions. The novelty of this study lies not in claiming that the NaOH–PVA combination has never been reported, but in evaluating a defined sequential strengthening route involving particle shaping, alkaline treatment, washing, drying, and PVA modification, followed by cementitious matrix optimization with silica fume and a polycarboxylate-based water-reducing agent. This approach is evaluated across two material systems, namely recycled aggregate concrete and high-content recycled aggregate cement-stabilized materials, to examine its effects on aggregate properties, mechanical performance, freeze–thaw resistance, and drying shrinkage.

## 2. Composition and Physical Properties of Recycled Aggregates

### 2.1. Composition and Chemical Characteristics of Recycled Aggregates

#### 2.1.1. Composition of Recycled Aggregates

**Figure 2:**
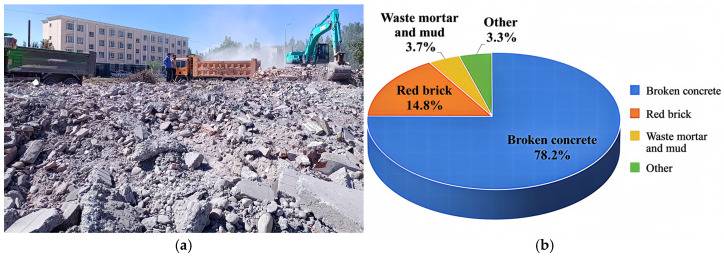
Source and composition of recycled aggregates derived from construction and demolition waste: (**a**) construction and demolition waste stockpile; (**b**) compositional distribution of recycled aggregates.

Recycled aggregates derived from construction and demolition waste usually exhibit complex and heterogeneous compositions, mainly including natural aggregate particles, residual mortar, crushed concrete, red brick fragments, ceramic particles, scrap metal, plastics, wood chips, and other impurities [3,6,35]. In this study, the recycled aggregates were obtained from solid waste generated during the demolition of concrete-structure buildings in Aksu, Xinjiang, China, as shown in Figure 2a. The coarse aggregates in the original concrete were mainly local pebbles, which are commonly used for concrete production in this region.

Before preparing recycled coarse aggregates, large visible impurities, such as scrap iron, plastics, and wood chips, were manually removed from the construction waste. The remaining waste concrete was then crushed and screened to obtain recycled coarse aggregates with the required particle size range. To determine the compositional distribution of the recycled aggregates, representative samples were collected from the aggregate stockpile using a multi-point sampling method. The collected samples were manually sorted according to visible material type, including natural aggregate, residual mortar, brick and ceramic fragments, and other minor impurities. Each component was weighed separately, and its mass percentage was calculated based on the total mass of the sorted sample.

#### 2.1.2. Chemical Composition Analysis

The chemical composition of the recycled coarse aggregate was determined using X-ray fluorescence spectrometry. Before the test, representative recycled aggregate samples were dried, crushed, and ground into fine powder to reduce the influence of particle heterogeneity on the test results. The prepared powder sample was then subjected to XRF analysis to determine the contents of the main oxide components. The test results are listed in Table 1.

As shown in Table 1, the recycled coarse aggregate was mainly composed of CaO, SiO_2_, and Al_2_O_3_, with contents of 75.34%, 15.58%, and 6.25%, respectively. The relatively high CaO content indicates that the recycled aggregate contained a large amount of calcium-rich residual mortar and cement hydration products attached to the surface of the original aggregate. This result is consistent with the typical characteristics of recycled aggregates prepared from waste concrete. The presence of SiO_2_ is mainly attributable to the original natural aggregate and silicate phases in the cementitious materials. In addition, Al_2_O_3_ may be related to cement hydration products, clay minerals, or minor brick and ceramic components.

In addition, the contents of Fe_2_O_3_, SO_3_, MgO, Na_2_O, K_2_O, and BaO were relatively low, accounting for 1.38%, 0.26%, 0.45%, 0.25%, 0.31%, and 0.18%, respectively. Under the present sampling and testing conditions, no obvious enrichment of sulfate or alkali oxide components was observed in the recycled coarse aggregate. Overall, the chemical composition indicates that the recycled aggregate retained typical cement-based characteristics, especially the presence of calcium-rich residual mortar. These characteristics may influence its pore structure, water absorption, density, and mechanical resistance, and provide a material basis for the subsequent alkaline treatment, PVA modification, and cementitious matrix optimization.

### 2.2. Physical and Mechanical Properties of Recycled Coarse Aggregates

According to the Chinese standard *Recycled Coarse Aggregate for Concrete* (GB/T 25177-2010), recycled coarse aggregates are defined as aggregates with particle sizes ranging from 4.75 mm to 40 mm that are obtained from waste concrete, masonry, and other construction waste through sorting, crushing, and screening processes [36]. Based on their physical and mechanical properties, recycled coarse aggregates are classified into Class I, Class II, and Class III. In addition, the industry standard *Technical Specification for Application of Recycled Aggregate* (JGJ/T 240-2011) provides recommended application ranges and technical requirements for different grades of recycled aggregates [37]. The classification criteria and corresponding application scope are summarized in Table 2.

To evaluate the basic quality of the recycled coarse aggregates used in this study, three key indicators were tested: water absorption, crushing index, and apparent density. These properties are closely related to the amount of attached old mortar, pore structure, internal microcracks, and mechanical resistance of recycled aggregates. The following subsections describe the specimen preparation procedures, testing conditions, calculation methods, and interpretation of results.

#### 2.2.1. Water Absorption Rate

Water absorption is an important indicator reflecting the pore structure and moisture uptake capacity of aggregates. For recycled coarse aggregates, it is closely associated with the amount of attached old mortar, open pores, and microcracks generated during the crushing process [12,38]. A high water absorption rate may affect the effective water-to-binder ratio, workability, and mechanical properties of recycled aggregate concrete [13,39].

In this study, large waste concrete slabs were first crushed and sorted using a CP-260 × 350 hammer crusher in the Civil Engineering Materials Laboratory. Particles larger than 31.5 mm were subjected to secondary crushing to ensure that the aggregate particle size satisfied the test requirements. Recycled coarse aggregates with particle sizes of 4.75–31.5 mm were selected for the water absorption test. Before testing, the aggregates were washed to remove surface dust, loose particles, and fine impurities attached to their surfaces.

The water absorption test was conducted in accordance with GB/T 25177-2010 and GB/T 14685-2011 [36,40]. Three parallel samples were prepared for each aggregate type, and each sample weighed approximately 5 kg. The aggregate samples were fully immersed in water at room temperature for 24 h. After immersion, the samples were removed from the water and gently wiped with a damp cloth until they reached a saturated surface-dry condition. The saturated surface-dry mass was then recorded immediately.

**Figure 3:**
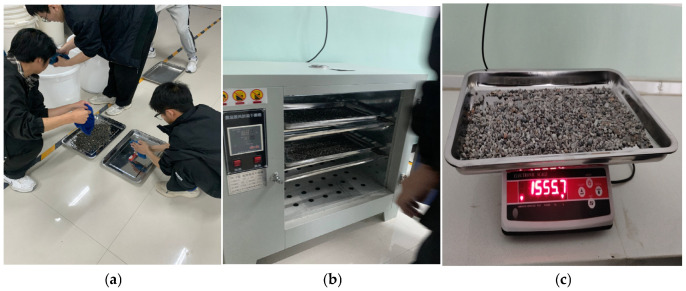
Experimental procedure for measuring the water absorption of recycled coarse aggregates: (**a**) immersion and surface-dry treatment; (**b**) oven drying at 105 ± 5 °C; (**c**) mass measurement after drying.

Subsequently, the samples were placed in a 101-2 electric blast drying oven manufactured by Beijing Hangtian Huayu Testing Instrument Co., Ltd., Beijing, China, and dried to constant mass at 105 ± 5 °C. After drying, the samples were cooled to room temperature and weighed immediately to minimize moisture exchange with the surrounding environment. The water absorption rate was calculated from the mass difference between the saturated surface-dry and oven-dry states. The testing procedure is shown in Figure 3.

The water absorption of the coarse aggregates was calculated using Equation (1):

(1) $$W_{a}=\frac{m_{1}-m_{0}}{m_{0}}\times 100\%$$

where *W_a_* is the water absorption of the aggregate (%); *m*_1_ is the mass of the saturated surface-dry aggregate (g); and *m*_0_ is the oven-dry mass of the aggregate (g).

The average value of three parallel tests was used as the final result. The results are shown in Table 3.

As shown in Table 3, the water absorption of the recycled coarse aggregate was 6.82%, which meets the requirement for Class III recycled aggregate specified in GB/T 25177-2010. In contrast, the water absorption of the natural coarse aggregate was only 1.38%, indicating that the recycled coarse aggregate had a much higher moisture absorption capacity.

This difference is mainly attributable to residual old mortar attached to the surface of the recycled aggregate. Compared with natural pebble aggregate, attached old mortar usually contains more capillary pores, microcracks, and weak interfacial transition zones. During the crushing process, additional microcracks may also be generated within the aggregate particles and at the interface between the natural aggregate and old mortar. These defects increase the accessible pore volume and provide more pathways for water penetration, resulting in higher water absorption [15,16]. From an engineering perspective, the high water absorption of recycled coarse aggregate should be considered in mixture design, particularly regarding pre-wetting treatment, additional mixing water, and workability control.

#### 2.2.2. Crushing Index

The crushing index is commonly used to evaluate the crushing resistance and mechanical stability of aggregates under compressive loading [36,40]. A lower crushing index indicates better resistance to crushing. In contrast, a higher crushing index suggests weaker load-bearing capacity and a greater possibility of particle breakage during concrete mixing, compaction, or service loading [7,9,13].

The crushing index test was conducted in accordance with GB/T 14685-2011 [40]. Recycled coarse aggregates with particle sizes of 4.75–31.5 mm were used for testing. Three parallel samples were prepared for each aggregate type, and each sample weighed approximately 3 kg. Each sample was placed into a standard cylindrical steel mold in two layers. After each layer was filled, the aggregate surface was leveled and compacted to achieve a dense and uniform sample. A pressure head was then installed, and the mold was carefully positioned at the center of the loading platen.

The aggregate crushing test was carried out using a YAW-300C microcomputer-controlled electro-hydraulic servo cement compression and flexure integrated testing machine, manufactured by Jinan Tianhua Testing Equipment Co., Ltd. (Jinan, China). During loading, the load was applied continuously and uniformly at 1 kN/s until it reached 200 kN, after which the maximum load was maintained for 5 s. After unloading, the sample was removed from the mold and sieved through a 2.36 mm sieve. The mass of the aggregate particles passing through the sieve was recorded. The crushing index was calculated using Equation (2), and the average value of the three parallel tests was taken as the final result.

(2) $$Q_{c}=\frac{m_{2}}{m_{1}}\times 100$$

where $Q_{c}$ is the crushing index of the aggregate (%); *m*_1_ is the original mass of the aggregate sample before loading (g); and *m*_2_ is the mass of particles passing through the 2.36 mm sieve after loading (g).

The crushing index values for the natural and recycled coarse aggregates obtained from the tests are listed in Table 4.

As shown in Table 4, the crushing index of the recycled coarse aggregate was 14.4%, which satisfies the Class II requirement of GB/T 25177-2010, namely not greater than 20%. However, this value was higher than that of the natural coarse aggregate, which had a crushing index of 5.2%. This result indicates that the recycled coarse aggregate had lower crushing resistance than the natural coarse aggregate.

The higher crushing index of the recycled coarse aggregate is mainly related to its composite structure. Recycled aggregate particles usually consist of the original natural aggregate and attached old cement mortar. The old mortar has lower strength and higher porosity than natural aggregate and is more likely to crack, peel off, or be crushed under compressive loading [11,41]. In addition, the crushing process used to prepare recycled aggregate may induce internal microcracks and interfacial defects. These defects act as stress concentration zones during loading, accelerating local fracture and particle breakage [12,16]. Therefore, although the recycled coarse aggregate meets the Class II requirement for the crushing index, its lower mechanical resistance should be considered when it is used in concrete with high replacement ratios.

#### 2.2.3. Apparent Density

Apparent density is defined as the mass per unit apparent volume of aggregate and is an important parameter for evaluating aggregate compactness and internal pore structure [36,40]. Compared with natural aggregates, recycled aggregates generally have a lower apparent density due to the presence of attached old mortar and internal defects.

**Figure 4:**
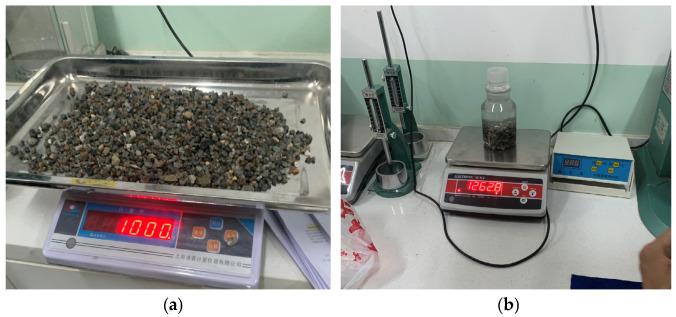
Determination of the apparent density of recycled aggregates using the wide-mouth bottle method: (**a**) weighing of recycled aggregate sample; (**b**) apparent density measurement using a wide-mouth bottle.

In this study, aggregate samples with particle sizes of 4.75–31.5 mm were prepared for the apparent density test. Two parallel samples were prepared for each aggregate type, and each sample weighed approximately 2 kg. The apparent density was determined using the wide-mouth bottle method in accordance with GB/T 14685-2011 [40]. Before testing, the aggregate samples were dried to constant mass at 105 ± 5 °C and then cooled to room temperature. The masses of the dry aggregate, the bottle filled with water, and the bottle containing aggregate and water were recorded in accordance with the standard procedure. The test process is shown in Figure 4.

The apparent density was calculated using Equation (3):

(3) $$\rho_{a}=\frac{m_{0}}{m_{0}+m_{2}-m_{1}}\times \rho_{w}-\alpha_{t}$$

where $\rho_{a}$ is the apparent density of the aggregate (kg/m^3^); *m*_0_ is the oven-dry mass of the aggregate sample (g); *m*_1_ is the total mass of the aggregate, bottle, water, and glass sheet (g); *m*_2_ is the total mass of the bottle, water, and glass sheet (g); $\rho_{w}$ is the density of water at the test temperature (kg/m^3^); and $\alpha_{t}$ is the water temperature correction coefficient specified in the standard.

The average of the two test results was used as the final result. If the difference between the two measured values exceeded 20 kg/m^3^, the test was repeated. The results are shown in Table 5.

As shown in Table 5, the apparent density of the recycled coarse aggregate was 2588 kg/m^3^, which satisfies the Class I requirement for apparent density specified in GB/T 25177-2010. The apparent density of the natural coarse aggregate was 2787 kg/m^3^. Although the apparent density of the recycled coarse aggregate was lower than that of the natural coarse aggregate, it still met the requirement for high-density recycled coarse aggregate.

The lower apparent density of the recycled coarse aggregate can be attributed to the presence of attached old cement mortar and the relatively loose internal structure of the recycled aggregate particles [11,38]. Old mortar generally has a lower density and higher porosity than natural aggregate. Moreover, microcracks generated during demolition and crushing further reduce the compactness of the recycled aggregate [12,41]. Therefore, the lower apparent density is consistent with the higher water absorption and weaker crushing resistance observed in the previous sections.

Based on the above test results, the recycled coarse aggregate used in this study met the Class III requirement for water absorption, the Class II requirement for crushing index, and the Class I requirement for apparent density according to GB/T 25177-2010. Considering that the water absorption was relatively high and did not meet the requirements for Class I or Class II recycled coarse aggregate, the untreated recycled coarse aggregate was comprehensively classified as Class III.

From an engineering application perspective, untreated Class III recycled coarse aggregate can generally be used for ordinary concrete with strength grades of C25 and below according to current specifications [36,37]. However, its relatively high water absorption and weaker crushing resistance indicate that the aggregate contains a considerable amount of attached old mortar, pores, and microcracks. These characteristics may adversely affect the workability, strength development, interfacial transition zone, and durability of recycled aggregate concrete, especially when high replacement ratios are adopted or when the concrete is exposed to freeze–thaw environments [31,32,33].

Therefore, appropriate strengthening treatments are necessary before using this recycled coarse aggregate in higher-performance concrete or concrete exposed to more demanding service conditions. Physical strengthening may remove weakly adhered mortar and improve particle morphology, while chemical or polymer modification may refine the pore structure, strengthen the interfacial transition zone, and reduce water absorption [27,28]. These considerations provide the experimental basis for the subsequent particle shaping, alkaline treatment, PVA modification, and cementitious matrix optimization conducted in this study.

## 3. Experimental Study on the Strengthening Technology of Recycled Coarse Aggregates

### 3.1. Raw Materials

All raw materials used in this study were inspected before testing to ensure compliance with the relevant standards and technical specifications.

The cementitious binder was sulfate-resistant Portland cement with a strength grade of 42.5. Natural river sand was used as the fine aggregate. The sand was classified as medium sand, with a fineness modulus of 2.3–3.0, and met the Class II sand requirements specified in the *Standard for Technical Requirements and Test Method of Sand and Crushed Stone for Ordinary Concrete* (JGJ 52-2006) [42]. Natural pebbles from the Aksu region, Xinjiang, China, were used as the natural coarse aggregate.

The recycled coarse aggregates were obtained from construction and demolition waste generated from demolished concrete structures, construction activities, and pavement removal in Aksu, Xinjiang, China. Before crushing, visible impurities, including brick fragments, wood chips, plastics, and metallic particles, were manually removed. The remaining waste concrete was then crushed and screened to obtain recycled coarse aggregates with a particle size range of 4.75–31.5 mm.

Sodium carbonate and sodium hydroxide were used for chemical strengthening. Sodium carbonate (Na_2_CO_3_) met the Class II requirements specified in *Industrial Sodium Carbonate and Its Test Methods–Part 1: Industrial Sodium Carbonate* (GB/T 210.1-2004) [43]. Sodium hydroxide (NaOH) complied with the requirements of *Industrial Sodium Hydroxide* (GB/T 209-2018) [44]. Polyvinyl alcohol (PVA-1788), supplied by Shanghai Jinshu, Shanghai, China, was used for polymer modification. A PC8020 polycarboxylate-based high-performance water-reducing agent was used, with a water-reduction rate of at least 25%, and its basic performance complied with the requirements of *Concrete Admixtures* (GB 8076-2008) [45]. Silica fume with a 300-mesh fineness and a specific surface area of at least 15,000 m^2^/kg was used to improve the compactness of the cementitious matrix and enhance the micro-filling effect [30,46]. Laboratory tap water from Xinjiang Institute of Technology was used for mixing and curing, and it met the requirements of the *Standard for Water Used in Concrete* (JGJ 63-2006) [47].

### 3.2. Preparation and Physical Strengthening of Recycled Coarse Aggregate

The recycled coarse aggregates were prepared from construction and demolition waste collected in Aksu, Xinjiang, China. Before crushing, visible impurities, including bricks, soil, wood fragments, plastics, metallic particles, and other non-concrete materials, were manually removed. The waste concrete was first crushed using a CP-260 × 350 hammer crusher. Particles larger than 31.5 mm were subjected to secondary crushing, and aggregates with particle sizes of 4.75–31.5 mm were selected by mechanical sieving. The selected aggregates were washed with tap water to remove surface dust, loose particles, and fine impurities. After washing, the aggregates were naturally drained and air-dried to a stable surface condition before subsequent physical strengthening and testing.

**Figure 5:**
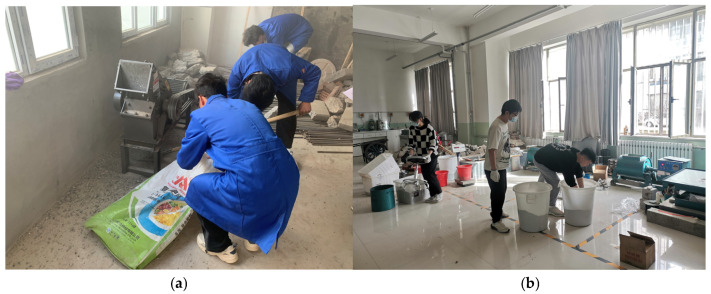
Preparation and physical strengthening of recycled coarse aggregates: (**a**) primary crushing using a CP-260 × 350 hammer crusher; (**b**) rolling strengthening using an HJW-30 forced concrete mixer.

The recycled coarse aggregates were physically strengthened using an HJW-30 single-horizontal-shaft forced concrete mixer manufactured by Beijing Hangtian Huayu Test Instrument Co., Ltd., Beijing, China, as shown in Figure 5. Before treatment, the inner wall and blades of the mixer were cleaned to avoid contamination by residual mortar and fine particles. For each batch, 20 kg of recycled coarse aggregate was placed in the mixer drum to maintain comparable rolling and abrasion conditions. The mixer was operated under dry conditions at a rotational speed of 48 r/min for 5 min. During rotation, repeated collisions, friction, and abrasion occurred among the aggregate particles and between the aggregates and the inner wall and blades of the mixer. This mechanical action removed part of the loose old mortar attached to the aggregate surface, rounded sharp edges, and improved particle morphology [19,20,21].

The physical strengthening process was controlled as a mild mechanical treatment to avoid excessive grinding. Excessive mechanical abrasion may induce additional microcracks in recycled aggregates, increase the amount of fine particles, and reduce aggregate quality. Therefore, after the rolling treatment, the aggregates were sieved again using a 4.75 mm square-hole sieve to remove detached mortar fragments and fine particles. The retained particle-shaped recycled coarse aggregates with particle sizes of 4.75–31.5 mm were used for subsequent alkaline treatment, PVA modification, physical property testing, and preparation of recycled aggregate concrete specimens. This process reduced aggregate heterogeneity and provided more uniform raw materials for subsequent strengthening and performance evaluation.

#### 3.2.1. Effect of Particle Shaping on Water Absorption and Crushing Index

The water absorption and crushing index of the recycled coarse aggregates before and after particle shaping are presented in Table 6. The water absorption of the untreated recycled coarse aggregate was 6.8%, whereas that of the particle-shaped recycled coarse aggregate decreased to 5.6%. The crushing index also decreased from 14.4% to 12.6% after particle shaping. These results indicate that particle shaping improved both the water absorption behavior and crushing resistance of the recycled coarse aggregate.

The improvement can be explained by the physical removal of weakly adhered mortar during rolling. Untreated recycled aggregates typically contain old cement mortar with high porosity, weak bond strength, and microcracks. These defects increase water absorption and reduce crushing resistance [12]. During particle shaping, part of the loose old mortar was detached and separated into the recycled fine-aggregate fraction, thereby reducing the content of porous mortar attached to the recycled coarse aggregate surface [19,20,21]. At the same time, repeated friction and impact among particles weakened sharp edges and removed weak surface layers. As a result, the aggregate surface became relatively denser and more stable, leading to lower water absorption and a lower crushing index.

However, the water absorption and crushing index of the particle-shaped recycled coarse aggregate were still higher than those of natural pebbles. This result suggests that physical strengthening can improve aggregate quality, but it cannot fully remove residual old mortar or internal defects. Therefore, further chemical or polymer modification remains necessary when recycled coarse aggregates are used at high replacement ratios in concrete [25,27,28].

#### 3.2.2. Effect of Particle Shaping on Bulk Density and Apparent Density

The effects of particle shaping on the bulk density and apparent density of recycled coarse aggregates are shown in Table 7. The bulk density of the recycled aggregate increased from 1326 kg/m^3^ to 1458 kg/m^3^ after particle shaping, representing a 9.96% increase. The apparent density increased from 2588 kg/m^3^ to 2678 kg/m^3^, corresponding to an increase of 3.48%. Nevertheless, the bulk density and apparent density of the particle-shaped recycled aggregate were still 9.2% and 3.9% lower than those of natural pebble aggregate, respectively.

The increase in density-related properties was mainly attributed to the removal of loose old mortar and the improvement of particle shape during the rolling treatment. Untreated recycled coarse aggregates generally have rough surfaces, angular edges, adhered mortar, internal microcracks, and residual cement paste particles. These characteristics increase the void content between particles and reduce both bulk density and apparent density. After particle shaping, part of the adhered porous mortar was removed, and the particle surface became relatively cleaner and more regular. Meanwhile, mechanical abrasion weakened sharp corners and improved particle roundness, thereby enhancing particle packing and reducing interparticle voids. These changes contributed to increases in bulk density and apparent density.

From a mechanical perspective, higher density generally indicates a lower content of weak porous phases and improved particle integrity. Therefore, the increase in density after particle shaping is consistent with the reduction in water absorption and crushing index reported in Table 6. Similar trends have also been observed in previous studies, in which mechanical grinding or particle shaping reduced the content of adhered mortar and improved the physical quality of recycled aggregates [19,20,21].

#### 3.2.3. Effect of Particle Shaping on the Compressive Strength of Recycled Aggregate Concrete

To evaluate the influence of particle shaping on the mechanical properties of recycled aggregate concrete, three types of concrete specimens were prepared using untreated recycled coarse aggregate, particle-shaped recycled coarse aggregate, and natural pebble aggregate. The corresponding mixtures were denoted as RCA, TRCA, and NA, respectively. The nominal water-to-cement ratio was fixed at 0.41 for all mixtures, as calculated from the water and cement contents of 175 kg/m^3^ and 427 kg/m^3^, respectively. To avoid combining mixture information with mechanical test results, the concrete mix proportions and compressive strength results are presented separately. The mix proportions of the three concrete groups are listed in Table 8.

For each concrete group, three 150 mm × 150 mm × 150 mm cubic specimens were prepared. Concrete mixing was performed using an HJW-30 single-horizontal-shaft forced concrete mixer. Before mixing, the mixer was cleaned and moistened to reduce the influence of residual materials and water absorption by the mixer wall. Coarse aggregate, medium sand, cement, and water were added sequentially according to the designed mix proportions, and the mixture was mixed until a uniform consistency was achieved. The fresh concrete was then placed into cube molds in layers and compacted to reduce entrapped air. After casting, the specimens were covered to prevent moisture loss and kept at room temperature for 24 h before demolding.

After demolding, the specimens were placed in an TB-40B standard constant-temperature and constant-humidity curing chamber, manufactured by Beijing Hangtian (Beijing, China). The curing temperature was maintained at 20 ± 3 °C, and the relative humidity was maintained at or above 95%. According to the Standard for *Test Methods of Physical and Mechanical Properties of Concrete* (GB/T 50081-2019), compressive strength tests were conducted after 7 and 28 days of curing [48]. The tests were performed using a YAW-3000 microcomputer-controlled automatic compression testing machine. Before loading, the bearing surfaces of the specimens were checked, and each specimen was aligned with the center of the lower platen to reduce eccentric loading. The load was applied continuously and uniformly until failure. The reported compressive strength values are the average results of three parallel specimens. The compressive strength results are summarized in Table 9.

The results show that particle shaping improved the compressive strength of recycled aggregate concrete. Compared with RCA concrete, the 7-day compressive strength of TRCA concrete increased from 10.3 MPa to 12.3 MPa, representing a 19.4% increase. The 28-day compressive strength increased from 14.3 MPa to 17.6 MPa, representing a 23.1% increase. These results indicate that the improvement in aggregate quality following particle shaping positively affected the strength development of recycled aggregate concrete.

The strength improvement can be attributed to the removal of some weakly adhered mortar and the reduction in surface defects during particle shaping. The cleaner and more regular aggregate surface may improve the contact conditions between recycled aggregate and new cementitious paste, thereby contributing to a denser interfacial transition zone [49]. However, the compressive strength of TRCA concrete was still lower than that of NA concrete, indicating that particle shaping alone could not eliminate the negative effects of residual old mortar, internal microcracks, and high porosity in recycled aggregates.

### 3.3. Alkali Treatment of Particle-Shaped Recycled Coarse Aggregate

To further improve the physical properties of the particle-shaped recycled coarse aggregate, alkaline treatment was conducted using sodium carbonate (Na_2_CO_3_) and sodium hydroxide (NaOH) solutions. The concentrations of the alkaline solutions were set as 2%, 3%, 4%, 5%, and 6% by mass, respectively. For each treatment condition, particle-shaped recycled coarse aggregates with a particle size range of 4.75–31.5 mm were immersed in the corresponding alkaline solution at room temperature for 24 h.

In this study, the mass ratio of alkaline solution to recycled coarse aggregate was fixed at 3:1. This ratio was selected to ensure complete immersion of the recycled coarse aggregates and to maintain a relatively stable alkaline environment during the 24 h soaking period. Based on the bulk density and apparent density of the particle-shaped recycled coarse aggregates, the inter-particle void content was estimated to be approximately 45%. Therefore, the adopted solution-to-aggregate ratio was sufficiently higher than the theoretical minimum required to fill the voids among the aggregate particles, which helped avoid local insufficient immersion. In addition, the relatively large solution volume reduced the influence of alkali consumption caused by reactions with the residual old mortar on the aggregate surface, thereby improving the comparability of different alkaline concentration groups. During immersion, the aggregates were gently stirred at regular intervals to improve the contact between the alkaline solution and the aggregate surface.

After immersion, the aggregates were removed from the solution and repeatedly washed with clean water until the pH of the rinsing water was close to neutral. This step was adopted to reduce the influence of residual alkali on subsequent physical property tests and concrete preparation. The washed aggregates were then spread in a thin layer and naturally air-dried under laboratory conditions until a stable surface condition was reached. The treated aggregates were stored in sealed containers before physical property testing and concrete specimen preparation.

The bulk density, apparent density, water absorption, and crushing index of the alkali-treated recycled coarse aggregates were measured in accordance with *Pebble and Crushed Stone for Construction* (GB/T 14685-2011) and *Recycled Coarse Aggregate for Concrete* (GB/T 25177-2010) [36,40]. For each treatment condition, repeated measurements were conducted according to the corresponding standard requirements, and the average values were used for analysis. This experimental design was adopted to evaluate the effects of alkaline solution type and concentration on the physical strengthening of particle-shaped recycled coarse aggregates.

The selection of Na_2_CO_3_ and NaOH was based on their different possible modification mechanisms. For Na_2_CO_3_ treatment, carbonate ions may react with calcium-bearing hydration products in the residual cement paste attached to the aggregate surface, promoting the formation of calcium carbonate (CaCO_3_). The generated CaCO_3_ may partially fill surface pores and microcracks, thereby improving the compactness of the adhered mortar layer and reducing water absorption [25,50]. However, the concentration of the Na_2_CO_3_ solution should be properly controlled. If the concentration is too low, the reaction may be insufficient; if it is too high, excessive surface reaction or crystallization may adversely affect the integrity of the adhered mortar layer.

**Figure 6:**
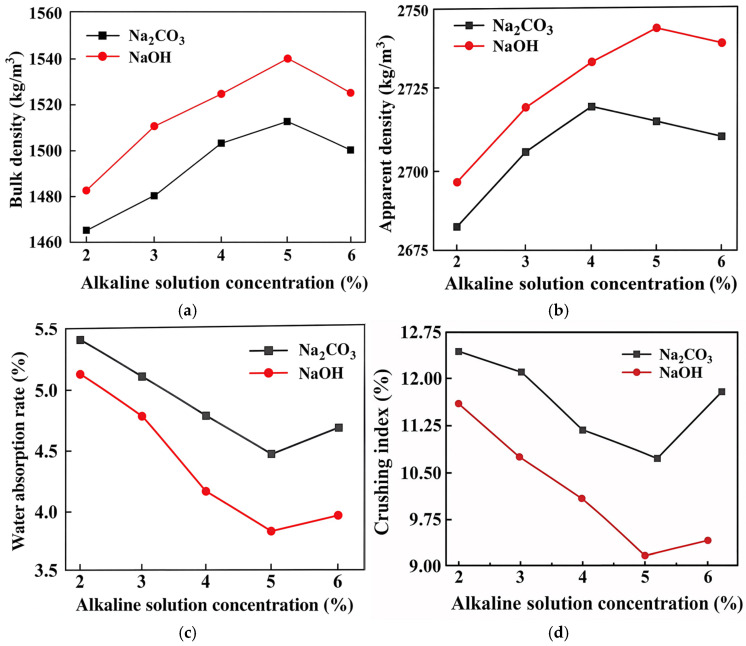
Effect of alkaline solution concentration on the physical properties of recycled coarse aggregates: (**a**) bulk density; (**b**) apparent density; (**c**) water absorption; (**d**) crushing index.

NaOH treatment provides a distinct modification pathway because of its high alkalinity. Under the present treatment conditions, the alkaline environment may alter weak surface products in the attached old mortar and modify the surface pore structure of recycled aggregates. However, the specific reaction products were not directly characterized in this study; therefore, the proposed explanation should be regarded as a possible interpretation of the observed changes in physical properties and concrete strength [26]. Considering the alkaline soil conditions commonly found in the Aksu region, as well as the need to compare different alkaline modification effects, the concentrations of the Na_2_CO_3_ and NaOH solutions were set at 2%, 3%, 4%, 5%, and 6%. The test results are shown in Figure 6.

According to the experimental results shown in Figure 6, the physical properties of recycled coarse aggregates were affected by both the type and concentration of the alkaline solution. As the solution concentration increased from 2% to 6%, the bulk density and apparent density of the recycled coarse aggregates first increased and then decreased slightly. The improvement was more evident at concentrations of 2–5%, while a slight deterioration was observed when the concentration further increased to 6%. This trend indicates that an appropriate alkaline concentration can improve the physical properties of recycled coarse aggregates, whereas excessive alkalinity may have an adverse effect.

For the Na_2_CO_3_-treated aggregates, the bulk density increased from 1465 kg/m^3^ at 2% concentration to 1512 kg/m^3^ at 5% concentration, representing a 3.2% increase. For the NaOH-treated aggregates, the bulk density increased from 1482 kg/m^3^ to 1540 kg/m^3^ over the same concentration range, representing a 3.9% increase. A similar trend was observed for apparent density. At a concentration of 5%, the apparent densities of the Na_2_CO_3_- and NaOH-treated aggregates reached 2715 kg/m^3^ and 2745 kg/m^3^, respectively, which were 1.3% and 1.85% higher than those obtained at 2% concentration. Compared with the particle-shaped recycled coarse aggregate before alkaline treatment, whose bulk density and apparent density were 1458 kg/m^3^ and 2678 kg/m^3^, respectively, both alkaline treatments further improved the density-related properties of the recycled aggregates.

The increase in density-related properties can be attributed to the modification of the porous old mortar attached to the recycled aggregate surface. For Na_2_CO_3_-treated aggregates, the possible formation of CaCO_3_ may help fill surface pores and microdefects, thereby increasing the compactness of the adhered mortar layer. For NaOH-treated aggregates, the strongly alkaline environment may promote the dissolution of weak surface phases and the formation of secondary cementitious products, thereby helping to densify the surface layer and reduce defects [50,51]. Compared with Na_2_CO_3_, NaOH showed a more pronounced improvement effect, which may be related to its higher alkalinity and stronger ability to modify loose hydration products and weak cementitious phases on the aggregate surface.

The water absorption and crushing index also changed with the increase in alkaline solution concentration. When the solution concentration increased from 2% to 5%, the water absorption of the Na_2_CO_3_-treated aggregates decreased from 5.4% to 4.5%, corresponding to a reduction of 16.7%. Meanwhile, the crushing index decreased from 12.4% to 10.8%, representing a reduction of 12.9%. The NaOH-treated aggregates exhibited a more obvious improvement. Their water absorption decreased from 5.1% to 3.9%, corresponding to a reduction of 23.5%, and the crushing index decreased from 11.6% to 9.3%, corresponding to a reduction of 19.8%.

These changes indicate that alkaline treatment can reduce the water absorption and improve the crushing resistance of recycled coarse aggregates. The reduction in water absorption is mainly associated with the refinement of surface pores and the decrease in connected capillary pores within the adhered old mortar. The decrease in crushing index suggests that the weak surface layer of recycled aggregates was partially removed or strengthened after alkaline treatment. In particular, NaOH treatment was more effective in improving aggregate quality, probably because OH^−^ ions can more readily interact with loose hydration products and promote surface reconstruction, thereby enhancing the compactness and mechanical integrity of the aggregate surface.

However, when the solution concentration increased to 6%, the density-related properties decreased slightly, while water absorption and crushing index increased to some extent. This result suggests that excessive alkalinity may adversely affect the aggregate structure. Excessive alkaline attack may damage the adhered mortar layer or induce additional microdefects, thereby weakening the beneficial pore-filling and surface-modification effects achieved at lower concentrations. Similar concentration-dependent effects have also been reported in previous studies on alkali-treated recycled aggregates [25,26].

Based on the above results, treatment with 5% NaOH solution was identified as the most effective alkaline strengthening method under the present experimental conditions. The recycled coarse aggregate treated with 5% NaOH exhibited a bulk density of 1540 kg/m^3^, an apparent density of 2745 kg/m^3^, a water absorption of 3.9%, and a crushing index of 9.3%. These values satisfy the technical requirements for Class II recycled coarse aggregates specified in *Recycled Coarse Aggregate for Concrete* (GB/T 25177-2010), namely water absorption not greater than 5.0%, crushing index not greater than 20%, and apparent density greater than 2350 kg/m^3^. Therefore, alkaline solution treatment, especially 5% NaOH treatment, can effectively improve the physical and mechanical properties of particle-shaped recycled coarse aggregates through surface modification, pore refinement, and strengthening of the adhered mortar layer. The 5% NaOH-treated recycled coarse aggregate was therefore selected for the subsequent PVA modification and concrete performance evaluation.

### 3.4. Effect of Alkali Treatment on the Compressive Strength of Recycled Aggregate Concrete

To evaluate the effect of alkaline treatment on the mechanical performance of recycled aggregate concrete, concrete specimens were prepared using particle-shaped recycled coarse aggregates treated with alkaline solutions at different concentrations. Untreated recycled aggregate concrete was used as the reference group, and natural aggregate concrete was used as the control group. The nominal water-to-cement ratio was fixed at 0.41 for all mixtures, as calculated from the water and cement contents of 175 kg/m^3^ and 427 kg/m^3^, respectively. This constant mixture design allowed the effect of alkaline treatment on compressive strength to be evaluated under comparable conditions.

Concrete cube specimens with dimensions of 150 mm × 150 mm × 150 mm were prepared in accordance with the Standard for *Test Methods of Concrete Physical and Mechanical Properties* (GB/T 50081-2019) [48]. Concrete mixing was conducted using an HJW-30 single-horizontal-shaft forced concrete mixer. Before mixing, the mixer was cleaned and moistened to reduce the influence of residual materials and avoid cross-contamination between different treatment groups. The aggregates were adjusted to a stable surface condition before mixing. Cement, medium sand, coarse aggregate, and water were weighed according to the designed mix proportions. The dry materials were first mixed to achieve a relatively uniform distribution, and water was then gradually added while mixing continued. The fresh concrete was cast into steel cube molds in layers and compacted to reduce entrapped air.

For each mixture and testing age, three replicate specimens were prepared. In total, 72 cube specimens were fabricated, including 36 specimens for 7-day testing and 36 specimens for 28-day testing. After casting, the specimens were covered to prevent moisture loss and kept in the molds for 24 h. They were then demolded and transferred to an SHBY-40B standard constant-temperature and constant-humidity curing chamber. The curing temperature was maintained at 20 ± 3 °C, with a relative humidity of at least 95%.

**Figure 7:**
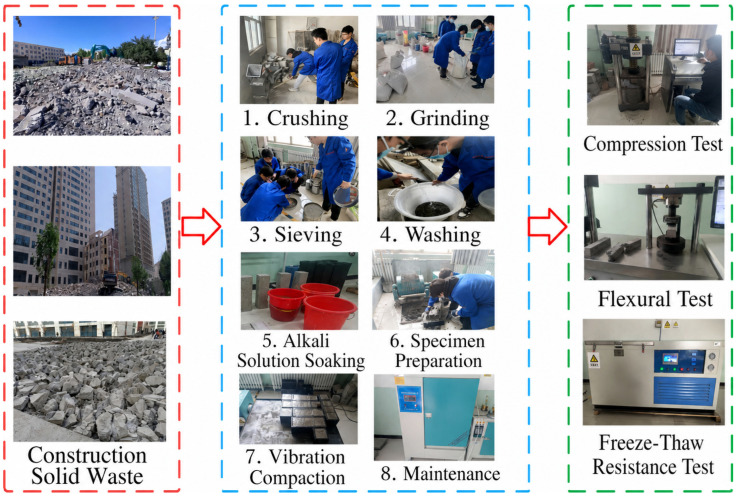
Flowchart of recycled aggregate concrete specimen preparation and performance testing using construction and demolition waste.

Compressive strength tests were conducted after 7 and 28 days of curing using a YAW-3000 microcomputer-controlled automatic compression testing machine. During testing, each specimen was centered on the lower platen to reduce eccentric loading, and the load was applied continuously and uniformly until failure. For each group, the arithmetic mean of the three measured values was taken as the representative compressive strength, with an accuracy of 0.1 MPa. The specimen preparation and testing procedure is shown in Figure 7.

In the specimen designations, Z represents concrete prepared with untreated recycled coarse aggregate, whereas T represents concrete prepared with natural aggregate. N-2 denotes concrete prepared with recycled coarse aggregate subjected to particle shaping followed by immersion in a 2% Na_2_CO_3_ solution, whereas NH-2 denotes concrete prepared with recycled coarse aggregate subjected to particle shaping followed by immersion in a 2% NaOH solution. The remaining designations follow the same naming rule: according to the corresponding alkaline solution concentration.

To address the concern that mixture compositions and mechanical properties should not be presented in the same table, the concrete mix proportions and compressive strength results are reported separately. The mix proportions of concrete prepared with alkali-treated recycled coarse aggregates are listed in Table 10, while the corresponding compressive strength results are summarized in Table 11.

As shown in Table 11, alkaline treatment improved the compressive strength of recycled aggregate concrete under the same nominal water-to-cement ratio and cement content. Compared with the untreated recycled aggregate concrete group Z, both Na_2_CO_3_ and NaOH treatments increased the 7-day and 28-day compressive strengths. Overall, the NaOH-treated groups, namely the NH series, exhibited higher compressive strengths than the Na_2_CO_3_-treated groups, namely the N series. The strength variation also showed a clear concentration-dependent trend.

For the Na_2_CO_3_-treated groups, the compressive strength increased as the Na_2_CO_3_ concentration increased from 2% to 4%. The 28-day compressive strength increased from 18.3 MPa for N-2 to 19.7 MPa for N-4. Compared with group Z, the 28-day compressive strength of N-4 increased by 36.8%. However, when the Na_2_CO_3_ concentration further increased to 5% and 6%, the 28-day compressive strengths were 19.5 MPa and 19.4 MPa, respectively, showing a slight decrease compared with N-4. This result indicates that the strengthening effect of Na_2_CO_3_ treatment tended to stabilize or slightly decline when the concentration exceeded 4%.

For the NaOH-treated groups, the compressive strength increased more markedly as the NaOH concentration increased from 2% to 5%. The 28-day compressive strength increased from 19.9 MPa for NH-2 to 24.1 MPa for NH-5, corresponding to a 21.1% increase. The NH-5 group achieved the highest strength among all alkali-treated recycled aggregate concrete groups, with 7-day and 28-day compressive strengths of 16.8 MPa and 24.1 MPa, respectively. Compared with group Z, the 7-day and 28-day compressive strengths of NH-5 increased by 64.7% and 67.4%, respectively. In addition, the 28-day compressive strength of NH-5 reached 85.2% of that of the natural aggregate concrete group T and was 23.6% higher than that of the N-5 group treated with the same concentration of Na_2_CO_3_ solution. These results suggest that 5% NaOH treatment was the most effective alkaline treatment condition under the present experimental conditions.

When the NaOH concentration further increased to 6%, the 28-day compressive strength decreased slightly from 24.1 MPa to 23.6 MPa, representing a reduction of 2.1% compared with NH-5. Although the strength of NH-6 remained higher than that of the untreated recycled aggregate concrete, the slight decrease indicates that increasing the alkaline concentration did not lead to continuous strength improvement. Therefore, the concentration of NaOH should be properly controlled during recycled aggregate modification.

The above results describe the observed strength variations caused by alkaline treatment at different concentrations. The following discussion interprets these trends in terms of surface mortar modification, pore refinement, and changes in the interfacial transition zone between the recycled aggregate and the new cementitious matrix.

The improvement in compressive strength may be associated with changes in the recycled aggregate surface and the interfacial transition zone. Recycled coarse aggregates usually contain adhered old mortar, microcracks, and connected pores, which lead to high water absorption and weak interfacial bonding [11,12,49]. After Na_2_CO_3_ treatment, carbonate ions may react with calcium-bearing hydration products in the old mortar and promote the possible formation of CaCO_3_. The generated CaCO_3_ may partially fill surface pores and microcracks, thereby increasing the compactness of the adhered mortar layer and reducing water absorption. This pore-filling effect may improve the mechanical integrity of recycled aggregates and enhance their bonding with the new cementitious matrix.

Compared with Na_2_CO_3_, NaOH has stronger alkalinity and may have a greater influence on the surface modification of recycled aggregates. OH^−^ ions may partially dissolve unstable silicate and aluminate phases in the old cement paste and remove weak surface products. The dissolved active components may further participate in secondary hydration reactions and contribute to the formation of additional cementitious products, such as calcium silicate hydrate gel. These products may fill pores, refine the surface pore structure, and improve the compactness of the aggregate surface. As a result, the interfacial transition zone between the NaOH-treated recycled aggregate and the new cementitious matrix may become denser, thereby improving stress transfer under compressive loading.

However, excessive alkaline concentration may have an adverse effect on the modified aggregate surface. When the NaOH concentration increased from 5% to 6%, the slight decrease in compressive strength may be related to over-etching of the adhered old mortar or the aggregate surface. Such excessive alkaline action could weaken the modified surface layer or induce additional surface defects, thereby reducing the beneficial effect of the treatment. This explains why the strength of NH-6 remained higher than that of group Z but was slightly lower than that of NH-5.

From the perspective of strength development, alkaline treatment improved both early-age and later-age compressive strengths. The 7-day strength of NH-5 reached 69.7% of its 28-day strength, which was close to that of group Z (70.8%). However, the absolute strength gain of NH-5 from 7 to 28 days was 7.3 MPa, higher than that of group Z (4.2 MPa). This result suggests that NaOH treatment improved the overall strength level of recycled aggregate concrete and contributed to continued strength development during curing. The later-age strength improvement may be related to a denser interfacial transition zone and a more favorable bonding condition around the modified recycled aggregate surface.

Nevertheless, the final strength of all recycled aggregate concrete groups remained lower than that of the natural aggregate concrete group. The 28-day compressive strength of NH-5 reached 85.2% of that of group T, whereas the corresponding value for group Z was only 50.9%. This indicates that 5% NaOH treatment can substantially narrow the strength gap between recycled aggregate concrete and natural aggregate concrete. However, the remaining difference also shows that alkaline treatment cannot fully offset the negative effects of residual old mortar, internal microcracks, and the relatively high porosity of recycled aggregates.

Therefore, based on the compressive strength results and the physical properties reported in Section 3.3, 5% NaOH treatment was selected as the preferred alkaline modification condition for the subsequent PVA modification and concrete performance evaluation. It should be noted that the present results mainly reflect the short-term compressive strength response under laboratory curing conditions. Further investigation is still needed to evaluate the durability, microstructural evolution, volume stability, and environmental impact of alkali-treated recycled aggregate concrete before large-scale engineering application.

### 3.5. Physical and Mechanical Properties of Recycled Coarse Aggregate Strengthened with Alkaline Solution and PVA

Polyvinyl alcohol (PVA) was introduced as a polymer modifier to further improve the physical and mechanical properties of recycled coarse aggregates after alkaline treatment. PVA has good film-forming and bonding characteristics and can potentially fill surface pores, seal microcracks, and improve the integrity of the aggregate surface [28,52]. After alkaline treatment modifies part of the weak old mortar attached to the recycled aggregate surface, subsequent PVA treatment may form a polymer film on the modified surface, thereby further reducing water absorption and improving the crushing resistance of recycled coarse aggregates.

Based on the results of Section 3.3 and Section 3.4, alkaline treatments with relatively effective concentrations were selected for subsequent PVA modification. The purpose of this section is to evaluate the combined effect of alkaline solution treatment and PVA modification on the physical and mechanical properties of particle-shaped recycled coarse aggregates and to determine a suitable composite treatment process. Considering the concentration-dependent behavior observed in the previous alkaline treatment tests, four composite treatment groups were designed: 4% Na_2_CO_3_ + 10% PVA, denoted as NP-4; 5% Na_2_CO_3_ + 10% PVA, denoted as NP-5; 4% NaOH + 10% PVA, denoted as NHP-4; and 5% NaOH + 10% PVA, denoted as NHP-5.

Previous studies on polymer-modified recycled aggregates have shown that polymer impregnation or coating can improve aggregate compactness, reduce water absorption, and enhance the mechanical performance of recycled aggregate concrete [27,28,29]. Based on these findings, PVA-1788, supplied by Shanghai Jinshu, China, was selected in this study. A 10% PVA solution was prepared by slowly adding PVA-1788 powder into water under continuous stirring to avoid agglomeration. The mixture was then heated to 80–95 °C and continuously stirred until the PVA powder was completely dissolved and a homogeneous solution was obtained. The solution was cooled to room temperature before use.

During the composite treatment process, the particle-shaped recycled coarse aggregates were first immersed in the corresponding alkaline solution for 24 h. The alkaline solution-to-aggregate mass ratio was fixed at 3:1 to ensure complete immersion of the aggregates and maintain a relatively stable alkaline environment during soaking. After alkaline immersion, the aggregates were removed from the solution and repeatedly washed with clean water until the pH of the rinsing water was close to neutral. This step was used to reduce the effect of residual alkali on the subsequent PVA treatment and concrete preparation. The washed aggregates were then naturally air-dried under laboratory conditions until no visible surface moisture remained.

The alkali-treated and air-dried aggregates were subsequently immersed in the 10% PVA solution for another 24 h, with the same solution-to-aggregate mass ratio of 3:1. This procedure allowed the polymer solution to contact the aggregate surface and penetrate some surface pores and microcracks. After PVA immersion, the aggregates were removed, drained, and naturally air-dried under laboratory conditions until a stable film formed on their surfaces. The treated aggregates were then stored in sealed containers before physical property testing and concrete specimen preparation.

The bulk density, apparent density, water absorption, and crushing index of the composite-treated recycled coarse aggregates were measured according to the relevant test methods specified in *Pebble and Crushed Stone for Construction* (GB/T 14685-2011) and *Recycled Coarse Aggregate for Concrete* (GB/T 25177-2010) [36,40]. For each composite treatment condition, repeated measurements were conducted according to the corresponding standard requirements, and the average values were used for analysis. The results are listed in Table 12.

As shown in Table 12, all alkaline solution–PVA composite treatments improved the physical and mechanical properties of recycled coarse aggregates compared with the untreated recycled aggregate. The bulk density and apparent density increased, while the water absorption and crushing index decreased. These results indicate that the composite treatment improved the compactness, surface quality, and crushing resistance of recycled coarse aggregates.

Among the different treatment methods, the NaOH-PVA composite treatment showed better overall performance than the Na_2_CO_3_-PVA treatment. For the NHP-5 group treated with 5% NaOH and 10% PVA, the apparent density reached 2779 kg/m^3^, which was 7.4% higher than that of the untreated recycled aggregate, 2588 kg/m^3^, and close to that of natural pebble aggregate, 2787 kg/m^3^. The bulk density of NHP-5 also increased to 1564 kg/m^3^, approaching that of natural pebble aggregate, 1606 kg/m^3^.

The water absorption of the NHP-5 group decreased from 6.8% to 3.3%, representing a reduction of 51.5% relative to the untreated recycled aggregate. Meanwhile, the crushing index decreased from 14.4% to 8.3%, corresponding to a reduction of 42.4%. These results show that the 5% NaOH + 10% PVA composite treatment substantially improved both the compactness and crushing resistance of recycled coarse aggregates.

For the Na_2_CO_3_-PVA composite treatment system, the NP-5 group also showed clear improvement. Its apparent density increased to 2728 kg/m^3^, representing a 5.4% increase compared with the untreated recycled aggregate. Its water absorption decreased from 6.8% to 4.0%, and the crushing index decreased from 14.4% to 10.2%, representing a 29.2% reduction. However, compared with the NHP-5 group, the improvement of NP-5 was less pronounced, indicating that the NaOH–PVA composite treatment was more effective in improving the physical and mechanical properties of recycled coarse aggregates.

The improvement achieved by the composite treatment can be explained by the possible complementary effects of alkaline solution modification and PVA film formation. Recycled coarse aggregates usually contain adhered old mortar, microcracks, and a porous surface layer. These defects increase water absorption and reduce crushing resistance. During alkaline treatment, NaOH or Na_2_CO_3_ may modify part of the old cement paste attached to the aggregate surface. For Na_2_CO_3_ treatment, carbonate ions may react with calcium-bearing hydration products and promote the formation of CaCO_3_, which can partially fill surface pores and improve the compactness of the adhered mortar layer. However, the modification depth and reactivity of Na_2_CO_3_ may be relatively limited.

Compared with Na_2_CO_3_, NaOH has stronger alkalinity and may more effectively modify the weak surface layer of recycled aggregates. OH^−^ ions may promote the dissolution of unstable hydration products and weak silicate or aluminate phases in the old mortar. This process can remove part of the loose surface products and provide a relatively cleaner and more reactive aggregate surface. The dissolved active components may also participate in secondary hydration reactions and contribute to the formation of additional cementitious products, such as calcium silicate hydrate gel. These products may fill pores, refine the pore structure, and improve the compactness of the recycled aggregate surface.

After alkaline treatment, PVA further contributes to the strengthening effect mainly through physical sealing and bonding. During PVA immersion, the polymer solution may enter surface pores and microcracks. After drying, PVA can form a continuous or semi-continuous polymer film on the aggregate surface. This film may seal capillary pores, reduce water penetration, and improve the integrity of the old mortar layer. Therefore, the decrease in water absorption is mainly related to pore blocking and surface film formation. Meanwhile, the reduction in crushing index may be associated with improved surface integrity and reduced development of microcracks under loading.

The better performance of the NHP-5 group indicates that NaOH treatment provided more favorable surface conditions for subsequent PVA modification. NaOH treatment may have modified part of the weak old mortar and improved the surface condition of the recycled aggregates, which was beneficial for PVA adsorption and film formation. The subsequent PVA coating further sealed the modified surface and reduced the connectivity of surface pores. This combined action improved the apparent density and crushing resistance of recycled aggregates more effectively than the Na_2_CO_3_-PVA treatment.

According to the technical requirements for Class II recycled coarse aggregates specified in *Recycled Coarse Aggregate for Concrete* (GB/T 25177-2010), the water absorption should not be greater than 5.0%, the crushing index should not be greater than 20%, and the apparent density should be greater than 2350 kg/m^3^. All composite-treated aggregates satisfied the requirements for Class II recycled coarse aggregates. In particular, the NHP-5 group exhibited the best overall performance, with an apparent density of 2779 kg/m^3^, water absorption of 3.3%, and crushing index of 8.3%.

From an engineering application perspective, reduced water absorption is beneficial for improving the stability of the effective water-to-cement ratio in recycled aggregate concrete. A lower crushing index indicates better aggregate resistance to mechanical damage during mixing and loading. Therefore, the combined treatment of 5% NaOH and 10% PVA can improve the applicability of recycled coarse aggregates in concrete production. However, the water absorption and crushing index of the NHP-5 group were still higher than those of natural pebble aggregate, indicating that the composite treatment cannot fully offset the influence of residual old mortar and internal microcracks.

Therefore, under the present experimental conditions, the 5% NaOH + 10% PVA treatment was identified as the preferred composite strengthening method for recycled coarse aggregates. Nevertheless, further research is still needed to evaluate the long-term durability of concrete prepared with NaOH-PVA-treated recycled aggregates, including chloride-ion permeability, freeze–thaw resistance, drying shrinkage, alkali-related expansion, and the long-term stability of the PVA film in the alkaline cementitious environment [31,32,33,34].

### 3.6. Effect of Composite Modification on the Compressive Strength of Recycled Aggregate Concrete

Based on the test results presented in Table 12, four groups of composite-modified recycled coarse aggregates were selected for concrete preparation: 4% Na_2_CO_3_ + 10% PVA, denoted as NP-4; 5% Na_2_CO_3_ + 10% PVA, denoted as NP-5; 4% NaOH + 10% PVA, denoted as NHP-4; and 5% NaOH + 10% PVA, denoted as NHP-5. The untreated recycled aggregate concrete, group Z, and the natural aggregate concrete, group T, were used as reference groups for comparative analysis, and their compressive strength values were taken from Table 11.

Concrete cube specimens with dimensions of 150 mm × 150 mm × 150 mm were prepared in accordance with the Standard for *Test Methods of Concrete Physical and Mechanical Properties* (GB/T 50081-2019) [48]. Concrete mixing was carried out using an HJW-30 single-horizontal-shaft forced concrete mixer. For all mixtures, the nominal water-to-cement ratio was fixed at 0.41, and the cement content was maintained at 427 kg/m^3^. This constant mixture design was adopted to ensure that the influence of composite aggregate modification on compressive strength could be evaluated under comparable conditions.

Before mixing, the mixer was cleaned and moistened to reduce the influence of residual materials and avoid cross-contamination between different treatment groups. The composite-modified recycled coarse aggregates were adjusted to a stable surface condition and weighed according to the designed mix proportions. Cement, medium sand, coarse aggregate, and water were then mixed until a relatively uniform concrete mixture was obtained. The fresh concrete was placed into steel cube molds in layers and compacted to reduce entrapped air and improve specimen uniformity. The mix proportions of concrete prepared with composite-modified recycled coarse aggregates are listed in Table 13.

After casting, the specimens were covered to prevent moisture loss and kept in molds for 24 h. They were then demolded and placed in an SHBY-40B standard constant-temperature and constant-humidity curing chamber. The curing temperature was maintained at 20 ± 3 °C, and the relative humidity was maintained at or above 95%. Compressive strength tests were conducted after 7 and 28 days of curing using a YAW-3000 microcomputer-controlled automatic compression testing machine. During testing, each specimen was centered on the lower platen to reduce eccentric loading, and the load was applied continuously and uniformly until failure.

For each mixture and testing age, three replicate cube specimens were prepared, giving a total of 24 specimens for the four composite-modified groups, including 12 specimens for 7-day testing and 12 specimens for 28-day testing. The arithmetic mean of the three replicate measurements was used as the representative compressive strength. According to the applicable test procedure, an individual result affected by abnormal failure or deviating from the mean by more than 15% would be considered invalid. In the available test records for this section, no exclusion was documented. Because the retained records mainly include calculated mean values, individual specimen results, standard deviations, coefficients of variation, and error bars are not reported for this test series. This limitation is further acknowledged in the Conclusions. The compressive strength results are listed in Table 14.

As shown in Table 14, the alkaline solution–PVA composite treatment improved the compressive strength of recycled aggregate concrete. Among the four composite-modified groups, the NHP-5 group, treated with 5% NaOH and 10% PVA, exhibited the highest compressive strength. Its 7-day and 28-day compressive strengths reached 18.2 MPa and 25.7 MPa, respectively. Compared with the untreated recycled aggregate concrete group Z, whose 28-day compressive strength was 14.4 MPa, the 28-day compressive strength of NHP-5 increased by 78.5%. Moreover, the 28-day strength of NHP-5 reached 90.8% of that of the natural aggregate concrete group T, whose 28-day strength was 28.3 MPa.

The other composite-modified groups also showed different degrees of strength improvement. The 28-day compressive strengths of NP-4, NP-5, and NHP-4 were 20.8 MPa, 20.6 MPa, and 23.8 MPa, respectively, corresponding to 73.5%, 72.8%, and 84.1% of the strength of natural aggregate concrete. These results indicate that the NaOH–PVA composite treatment was more effective than the Na_2_CO_3_–PVA composite treatment in improving compressive strength. In particular, the 28-day strength of NHP-5 was 24.8% higher than that of NP-5, indicating the better strengthening efficiency of the NaOH-based composite treatment.

The comparison between NP-4 and NP-5 shows that increasing the Na_2_CO_3_ concentration from 4% to 5% did not further improve the 28-day compressive strength. The 28-day strength slightly decreased from 20.8 MPa to 20.6 MPa. This result suggests that the strengthening effect of the Na_2_CO_3_–PVA treatment tended to stabilize when the Na_2_CO_3_ concentration exceeded 4%. This trend is consistent with the single alkaline treatment results in Table 11, where the Na_2_CO_3_-treated groups also showed limited strength improvement at relatively high concentrations.

In contrast, the NaOH–PVA system showed a more obvious concentration-dependent improvement. When the NaOH concentration increased from 4% to 5%, the 28-day compressive strength increased from 23.8 MPa to 25.7 MPa, corresponding to an 8.0% increase. The above comparisons mainly describe the observed strength variations among the composite-modified groups. The following discussion further interprets these differences from the perspectives of aggregate surface modification, polymer film formation, and aggregate–matrix interfacial bonding. The relatively higher strength of the 5% NaOH + 10% PVA group may be attributed to a more favorable aggregate surface condition for subsequent PVA modification and improved bonding between the recycled aggregate and the cementitious matrix.

The improved strength of the NaOH–PVA-modified recycled aggregate concrete may be attributed to the combined effect of alkaline modification and polymer film formation. Recycled coarse aggregates usually contain adhered old mortar, surface pores, and microcracks, which weaken the interfacial transition zone between the aggregate and the new cementitious matrix. NaOH treatment may partially dissolve or remove weak hydration products and loose old mortar from the aggregate surface. It may also activate some silicate and aluminate phases in the old cement paste, thereby contributing to the formation of additional hydration products. These effects can help refine the surface pore structure and improve the compactness of the aggregate surface.

After NaOH treatment, PVA further modifies the aggregate surface mainly through physical sealing and bonding. During immersion, the PVA solution may penetrate some surface pores and microcracks. After drying, a continuous or semi-continuous polymer film can form on the aggregate surface, reducing water absorption and blocking connected pores. This polymer film may also improve the bonding condition between the recycled aggregate and the cementitious matrix. As a result, the interfacial transition zone may become denser, stress transfer under compressive loading may become more effective, and the compressive strength of recycled aggregate concrete is improved.

Compared with NaOH, Na_2_CO_3_ has relatively weaker alkalinity and a lower ability to modify the weak old mortar layer. Although carbonate ions may react with calcium-containing hydration products to form CaCO_3_, thereby partially filling surface pores and improving compactness, the modification effect of Na_2_CO_3_ is mainly associated with surface precipitation and pore filling. Therefore, the Na_2_CO_3_–PVA system also improved the compressive strength of recycled aggregate concrete, but its strengthening effect was weaker than that of the NaOH–PVA system.

From an engineering application perspective, the NHP-5 group restored the 28-day compressive strength of recycled aggregate concrete to more than 90% of that of natural aggregate concrete. This indicates that the combined treatment with 5% NaOH and 10% PVA can effectively reduce the strength gap between recycled aggregate concrete and natural aggregate concrete under the present experimental conditions. Such an improvement is meaningful for enhancing the application potential of recycled coarse aggregates in concrete production, particularly at high replacement levels. However, the strength of NHP-5 remained lower than that of natural aggregate concrete, indicating that residual old mortar, internal microcracks, and the long-term stability of the polymer-modified surface may continue to influence the performance of recycled aggregate concrete.

Overall, the combined treatment with 5% NaOH and 10% PVA was the most effective modification method among the tested schemes. This treatment improved the compressive strength of recycled aggregate concrete and provided a feasible technical parameter for the preparation of strengthened recycled coarse aggregates. Nevertheless, further studies are still needed to evaluate the durability performance of concrete prepared with NaOH–PVA-modified recycled aggregates, including chloride-ion permeability, freeze–thaw resistance, drying shrinkage, alkali-related expansion, and the long-term stability of the PVA film in the highly alkaline cementitious environment.

### 3.7. Combined Strengthening of Recycled Coarse Aggregate and Cementitious Matrix

#### 3.7.1. Effect of Combined Aggregate–Matrix Strengthening on Compressive Strength

To further improve the mechanical performance of recycled aggregate concrete, silica fume was incorporated into the cementitious matrix as a supplementary cementitious material. Silica fume has an extremely fine particle size, generally ranging from 0.1 to 0.3 μm, which is much smaller than that of ordinary cement particles. Owing to its fine particle size, silica fume can fill microvoids and capillary pores in the cementitious matrix, thereby improving the compactness of cement paste and the interfacial transition zone (ITZ) between recycled coarse aggregate and cement paste. In addition, the amorphous SiO_2_ in silica fume can react with calcium hydroxide (CH) generated during cement hydration to form additional calcium silicate hydrate (C-S-H) gel. This pozzolanic reaction may contribute to pore refinement, matrix densification, and improved aggregate–matrix bonding [30,46,49,53].

However, excessive silica fume may reduce concrete workability because of its high specific surface area and increased water demand. Accordingly, a polycarboxylate-based water-reducing agent was introduced to improve workability without increasing the mixing water content. Based on preliminary mix optimization and previous studies on cementitious matrix modification, the adopted strengthening scheme consisted of 10% silica fume and 0.2% polycarboxylate-based water-reducing agent by mass of cement [30,45,46,54,55].

Based on the results presented in Section 3.5 and Section 3.6, four groups of composite-modified recycled coarse aggregates were selected for subsequent cementitious matrix optimization: 4% Na_2_CO_3_ + 10% PVA, 5% Na_2_CO_3_ + 10% PVA, 4% NaOH + 10% PVA, and 5% NaOH + 10% PVA. The corresponding aggregate groups were denoted as NP-4, NP-5, NHP-4, and NHP-5, respectively. These composite-modified recycled coarse aggregates were then used to prepare recycled aggregate concrete with silica fume and a polycarboxylate-based water-reducing agent. The purpose of this part was to evaluate whether simultaneous strengthening of recycled coarse aggregate and cementitious matrix could further improve the compressive strength of recycled aggregate concrete.

Concrete cube specimens with dimensions of 150 mm × 150 mm × 150 mm were prepared according to GB/T 50081-2019 [48]. The composite-modified recycled coarse aggregates, medium sand, cement, silica fume, water, and polycarboxylate-based water-reducing agent were weighed according to Table 15. For all recycled aggregate concrete mixtures, the nominal water-to-cement ratio was maintained at 0.41, silica fume was added at 10% of the cement mass, and the water-reducing agent was added at 0.2% of the cement mass. The specimens were demolded after 24 h, cured at 20 ± 3 °C and a relative humidity of at least 95%, and tested for compressive strength at 7 and 28 days.

Three replicate specimens were prepared for each mixture and each testing age. To reduce the influence of experimental variability, the arithmetic mean of the three measured values was used as the representative compressive strength. If the strength value of an individual specimen deviated from the mean value by more than 15%, it was excluded, and the arithmetic mean of the remaining valid results was used as the final compressive strength. In this section, no individual result exceeded this 15% deviation criterion; therefore, all measured values were retained for calculating the reported averages.

To improve the organization and readability of the experimental data, the mix proportions and compressive strength results are reported separately. The mix proportions of concrete with combined strengthening of recycled coarse aggregate and cementitious matrix are listed in Table 15, and the corresponding compressive strength results are presented in Table 16.

As shown in Table 16, incorporating 10% silica fume and 0.2% polycarboxylate-based water-reducing agent further improved the compressive strength of recycled aggregate concrete prepared with composite-modified recycled coarse aggregates. Among all modified groups, the NHP-5 group, treated with 5% NaOH + 10% PVA and subsequently modified with silica fume and a water-reducing agent, exhibited the best mechanical performance. Its 7-day and 28-day compressive strengths reached 19.9 MPa and 27.6 MPa, respectively. Compared with the natural aggregate concrete group T, whose 7-day and 28-day compressive strengths were 21.3 MPa and 28.3 MPa, the strengths of NHP-5 reached 93.4% and 97.5% of the corresponding values of natural aggregate concrete, respectively.

The other modified groups also showed different degrees of strength improvement. The 7-day and 28-day compressive strengths of NP-4 were 15.2 MPa and 24.5 MPa, corresponding to 71.4% and 86.6% of those of natural aggregate concrete. The NP-5 group reached 16.5 MPa and 25.3 MPa, corresponding to 77.5% and 89.4%, respectively. The NHP-4 group reached 17.6 MPa and 26.2 MPa, corresponding to 82.6% and 92.6%, respectively. These results indicate that the NaOH–PVA composite modification was more effective than the Na_2_CO_3_–PVA composite modification in improving the compressive strength of recycled aggregate concrete.

Compared with the untreated recycled aggregate concrete group Z, whose 28-day compressive strength was 14.4 MPa, the 28-day compressive strength of NHP-5 increased by 91.7%. Compared with the NHP-5 concrete without silica fume and water-reducing agent modification, whose 28-day compressive strength was 25.7 MPa, the matrix-modified NHP-5 concrete further increased to 27.6 MPa. This result indicates that strengthening the recycled coarse aggregate alone can considerably improve concrete strength. Moreover, additional optimization of the cementitious matrix can further reduce the strength gap between recycled aggregate concrete and natural aggregate concrete.

The strength improvement may be attributed to the combined effect of aggregate surface strengthening and cementitious matrix densification. In recycled aggregate concrete, adhered old mortar, surface pores, and microcracks in recycled aggregates usually weaken the ITZ and reduce the load-transfer efficiency between the aggregate and the new cement paste [11,12,49]. NaOH treatment may remove or modify part of the weak residual mortar, thereby improving the compactness and surface condition of recycled coarse aggregates. PVA treatment can further seal some surface pores and microcracks through film formation, which may improve the integrity of the recycled aggregate surface and the bonding condition between the aggregate and the cementitious matrix.

In addition to aggregate modification, silica fume may improve the cementitious matrix through micro-filling and pozzolanic effects. Fine silica fume particles can fill capillary pores and voids in the cement paste and ITZ. Meanwhile, the pozzolanic reaction between silica fume and calcium hydroxide may generate additional C-S-H gel, reducing the amount of weak CH crystals and improving the compactness of the hardened cement paste. The polycarboxylate-based water-reducing agent improves particle dispersion and mixture workability, which helps reduce local defects caused by insufficient compaction. Therefore, under the present experimental conditions, the combined use of NaOH, PVA, silica fume, and the water-reducing agent may improve the aggregate surface, ITZ, and cementitious matrix simultaneously.

From an engineering perspective, the 28-day compressive strength of the NHP-5 group reached 97.5% of that of natural aggregate concrete, which is a notable improvement under the present experimental conditions. This suggests that recycled coarse aggregates treated with 5% NaOH + 10% PVA, combined with 10% silica fume and 0.2% polycarboxylate-based water-reducing agent in the cementitious matrix, can produce recycled aggregate concrete with mechanical performance close to that of natural aggregate concrete. This provides a feasible technical route for improving the utilization potential of recycled coarse aggregates in concrete production, especially when a high replacement level of recycled coarse aggregate is adopted.

However, although the compressive strength approached that of natural aggregate concrete, this result should not be interpreted as complete equivalence between recycled aggregate concrete and natural aggregate concrete. Residual old mortar, internal microcracks, and the higher porosity of recycled aggregates may still affect long-term performance. In addition, the stability of the PVA film in the highly alkaline cementitious environment requires further verification. Therefore, long-term durability, volume stability, chloride-ion permeability, freeze–thaw resistance, drying shrinkage, alkali-related expansion, and microstructural evolution should be further investigated before large-scale engineering application.

Overall, under the present experimental conditions, the combined strengthening scheme of 5% NaOH + 10% PVA for recycled coarse aggregate, together with 10% silica fume and 0.2% polycarboxylate-based water-reducing agent for the cementitious matrix, achieved the best improvement among the tested schemes. This method effectively enhanced the compressive strength of recycled aggregate concrete and provided a practical basis for the engineering application of strengthened recycled coarse aggregates.

#### 3.7.2. Microstructural Characteristics of Concrete with Combined Strengthening of Recycled Coarse Aggregate and Cementitious Matrix

To further examine the influence of the combined strengthening method on the microstructure of recycled aggregate concrete, scanning electron microscopy (SEM) observations were conducted on specimens subjected to different strengthening treatments. SEM analysis was performed using a ZEISS Sigma 300 scanning electron microscope manufactured by ZEISS, Jena, Germany. The specimens included concrete prepared with NaOH-treated recycled coarse aggregate, concrete prepared with 5% NaOH + 10% PVA-treated recycled coarse aggregate, and concrete prepared with 5% NaOH + 10% PVA-treated recycled coarse aggregate combined with 10% silica fume and 0.2% polycarboxylate-based water-reducing agent.

**Figure 8:**
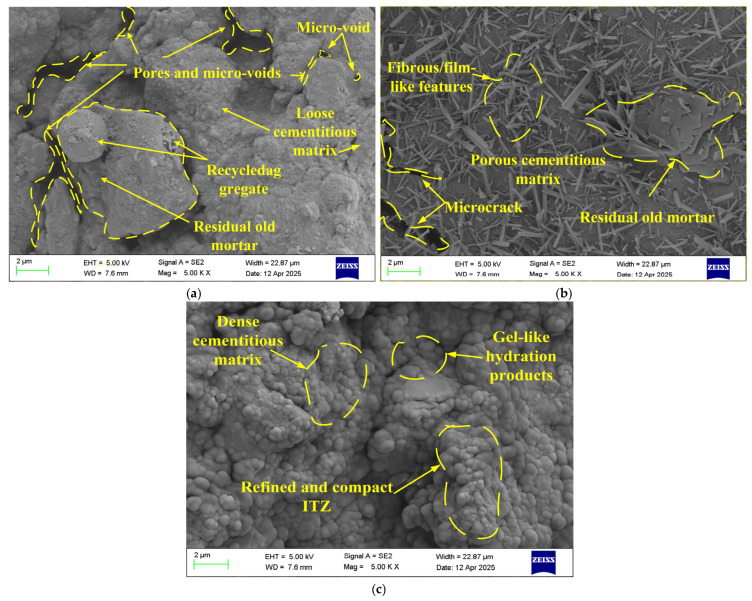
SEM micrographs of recycled aggregate concrete under different strengthening treatments: (**a**) control specimen prepared with NaOH-treated recycled aggregate; (**b**) recycled aggregate concrete prepared with 5% NaOH + 10% PVA-treated recycled aggregate; (**c**) recycled aggregate concrete prepared with 5% NaOH + 10% PVA-treated recycled aggregate and a cementitious matrix modified with 10% silica fume and 0.2% polycarboxylate-based water-reducing agent.

Before SEM observation, representative fragments were collected from the fractured surfaces of compressive strength specimens after 28 days of standard curing, particularly from regions near the recycled aggregate–cementitious matrix interface. Loose particles and surface dust were carefully removed, and the samples were dried prior to observation. The interfacial transition zone (ITZ) between the recycled coarse aggregate and the cementitious matrix was examined in detail, as it is generally regarded as a weak zone in recycled aggregate concrete. SEM observations were used to qualitatively compare the pore structure, microcrack distribution, hydration products, and bonding conditions at the aggregate–matrix interface after different strengthening treatments. Representative SEM micrographs are shown in Figure 8.

As shown in Figure 8a, the specimen prepared with NaOH-treated recycled coarse aggregate showed a relatively loose and discontinuous microstructure near the aggregate–matrix interface. Although NaOH treatment may partially modify the weak old mortar attached to the recycled aggregate surface, pores, microvoids, microcracks, and residual old mortar were still observed in the interfacial region. These defects may reduce the compactness of the ITZ and provide preferential paths for crack initiation and propagation under compressive loading. This observation is consistent with the compressive strength results, which showed that alkaline treatment alone improved the performance of recycled aggregate concrete. However, the improvement remained limited compared with the subsequent combined strengthening schemes.

After further treatment with 10% PVA, the microstructure near the ITZ became relatively denser, as shown in Figure 8b. During immersion, the PVA solution may penetrate surface pores and microcracks in the recycled aggregate. After drying, a film-like polymer phase may form on the aggregate surface, thereby improving surface integrity and reducing pore connectivity. Compared with the NaOH-treated specimen, fewer open pores and microcracks were observed around the aggregate–matrix interface. This indicates that PVA treatment helped improve the compactness of the recycled aggregate surface and the bonding condition between the recycled aggregate and the cementitious matrix. The microstructural refinement observed in Figure 8b is consistent with the increase in compressive strength of concrete prepared with NaOH–PVA-treated recycled aggregate.

For the specimen prepared with 5% NaOH + 10% PVA-treated recycled aggregate and a cementitious matrix modified with silica fume and a water-reducing agent, the microstructure was further refined, as shown in Figure 8c. The cementitious matrix appeared denser and more continuous, and the ITZ between the recycled aggregate and cementitious matrix became more compact. Gel-like hydration products were observed in the interfacial region, indicating that the local microstructure was refined. The fine silica fume particles may fill residual voids in the cementitious matrix and ITZ. Meanwhile, the pozzolanic reaction between silica fume and calcium hydroxide may promote the formation of additional gel-like hydration products, which can further fill capillary pores and improve the compactness of the hardened matrix. However, because the identification of hydration products based only on SEM morphology is limited, these products are described as gel-like hydration products rather than being definitively identified as a specific phase.

The SEM observations provide qualitative support for the compressive strength results listed in Table 16. The 28-day compressive strength of the NHP-5 group with silica fume and a water-reducing agent reached 27.6 MPa, approaching that of natural aggregate concrete. This improvement may be related to the combined effects of aggregate surface modification, polymer film formation, and cementitious matrix densification. NaOH treatment may improve the surface condition of recycled aggregate by modifying or removing part of the weak old mortar layer. PVA treatment may further seal surface pores and microcracks through film formation. Silica fume may refine the cementitious matrix and ITZ through micro-filling and pozzolanic reactions. Meanwhile, the polycarboxylate-based water-reducing agent may improve the dispersion of cementitious particles and help form a denser hardened matrix.

Overall, the SEM results indicate that the combined use of NaOH treatment, PVA modification, silica fume, and a polycarboxylate-based water-reducing agent improved the microstructural characteristics of recycled aggregate concrete. The aggregate surface became more compact, the ITZ was refined, pore connectivity was reduced, and the bonding condition between recycled aggregate and cementitious matrix was improved. These microstructural changes help explain the observed increase in compressive strength. Nevertheless, SEM observation is mainly qualitative and limited to selected local regions. Therefore, further quantitative characterization, such as mercury intrusion porosimetry, X-ray computed tomography, or backscattered electron image analysis, is still needed to verify pore structure refinement and ITZ densification more comprehensively.

## 4. Performance of High-Content Recycled Aggregate Cement-Stabilized Materials

To evaluate the engineering applicability of recycled coarse aggregates in cement-stabilized materials with high recycled aggregate contents, a systematic experimental investigation was conducted using recycled coarse aggregate replacement ratios of 50%, 60%, 70%, 80%, 90%, and 100%. Two types of recycled coarse aggregates were considered in this section. The NCR series was prepared with particle-shaped recycled coarse aggregate without NaOH–PVA composite treatment, whereas the CR series was prepared with recycled coarse aggregate treated sequentially with 5% NaOH and 10% PVA. The main performance indicators investigated included unconfined compressive strength, splitting tensile strength, compressive rebound modulus, frost resistance, and drying shrinkage performance.

It should be noted that the material system investigated in Section 4 is different from the recycled aggregate concrete studied in Section 3. Section 3 focused on recycled aggregate concrete prepared with a nominal water-to-cement ratio of 0.41, a cement content of 427 kg/m^3^, and 150 mm cubic specimens. In contrast, Section 4 focused on high-content recycled aggregate cement-stabilized materials with a cement dosage of 5% by mass of total dry aggregate, prepared by static compaction into Ø100 mm × 100 mm cylindrical specimens. Therefore, the optimized aggregate treatment obtained in Section 3, namely 5% NaOH + 10% PVA, was transferred to Section 4 to evaluate its applicability in a different cement-stabilized material system intended for pavement base or subgrade applications.

Before specimen preparation, the raw materials were screened and cleaned to remove visible impurities. Six RCA replacement ratios, from 50% to 100% at 10% intervals, were adopted to evaluate high-content recycled aggregate cement-stabilized materials. In the group designation, NCR denotes cement-stabilized materials containing particle-shaped RCA without NaOH–PVA composite treatment, whereas CR denotes cement-stabilized materials containing RCA treated sequentially with 5% NaOH and 10% PVA before specimen preparation. To clarify the material system, mixture compositions, and controlled preparation parameters used in Section 4, Table 17 summarizes the experimental design. The proportions were calculated for one standard cylindrical specimen (Ø100 mm × 100 mm). Because the NCR and CR mixtures had identical component quantities at each RCA replacement level and differed only in the RCA treatment condition, they are grouped in Table 17 for concise presentation. The cement dosage, aggregate fractions, molding water content, compaction parameters, and curing conditions are provided in the table note.

For the quantitative tests reported in Section 4, three replicate specimens were tested for each mixture and test condition. Unless a test result was invalidated because of abnormal specimen failure, eccentric loading, or an obvious testing deviation under the applicable test procedure, no individual result was excluded from the reported mean values. The reported values represent the arithmetic means of the available replicate measurements. Because individual measurements were not retained for several test series, standard deviations, coefficients of variation, individual data points, and error bars could not be reported comprehensively. This limitation is acknowledged in the Conclusions.

### 4.1. Unconfined Compressive Strength

**Figure 9:**
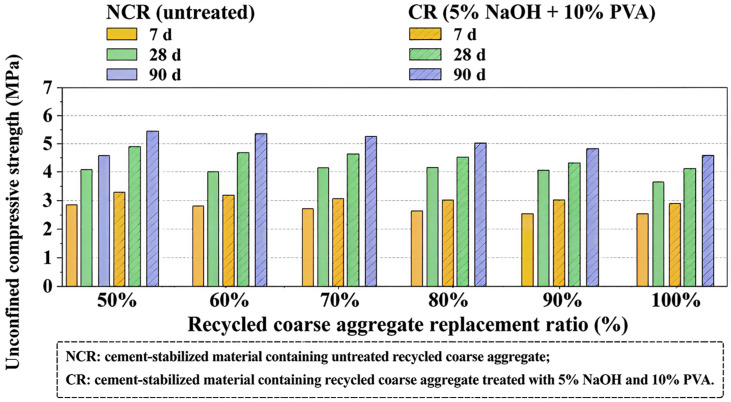
Average unconfined compressive strength of high-content recycled aggregate cement-stabilized materials with different recycled coarse aggregate replacement ratios at curing ages of 7, 28, and 90 days.

Twelve specimen groups were prepared to evaluate the effects of RCA replacement ratio and NaOH–PVA composite modification on the unconfined compressive strength of high-content recycled aggregate cement-stabilized materials. The RCA replacement ratios were 50%, 60%, 70%, 80%, 90%, and 100%, and the NCR and CR series were prepared according to the mixture compositions and controlled preparation parameters listed in Table 17. After curing at 20 ± 2 °C and a relative humidity of at least 95%, unconfined compressive strength was tested at 7, 28, and 90 days using a YAW-3000 compression testing machine in accordance with JTG E51-2009 [56]. The mean compressive strength values are presented in Figure 9.

As shown in Figure 9, the average unconfined compressive strength of both NCR and CR specimens increased with curing age. This indicates that the cementitious matrix continued to hydrate under standard curing conditions, resulting in the gradual development of strength. However, at the same curing age, the average strength generally decreased with increasing recycled coarse aggregate replacement ratio.

For the NCR specimens, the average unconfined compressive strength decreased with increasing recycled coarse aggregate content. At 7 days, the strength decreased from 2.81 MPa to 2.50 MPa as the replacement ratio increased from 50% to 100%, representing an 11.0% reduction. At 28 days, the strength decreased from 4.22 MPa to 3.55 MPa, corresponding to a reduction of 15.9%. At 90 days, the strength decreased from 4.72 MPa to 4.05 MPa, corresponding to a reduction of 14.2%. These results suggest that increasing the content of particle-shaped recycled coarse aggregate without NaOH–PVA composite treatment adversely affected the strength development of the cement-stabilized materials.

The reduction in strength is mainly attributed to the inherent defects that remained in the particle-shaped recycled coarse aggregates. Compared with natural aggregates, recycled coarse aggregates usually contain residual old mortar, surface pores, microcracks, and relatively weak interfacial regions. These defects increase the water absorption of aggregates and weaken the bond between recycled coarse aggregates and the new cementitious matrix. As the replacement ratio increases, the proportion of these defective aggregate particles also increases, leading to a gradual decrease in unconfined compressive strength.

For the CR specimens, the average unconfined compressive strength also decreased as the recycled coarse aggregate replacement ratio increased. However, the CR specimens exhibited higher average strength than the corresponding NCR specimens across all replacement ratios and curing ages. At 7 days, the strength of the CR specimens decreased from 3.25 MPa to 2.85 MPa as the replacement ratio increased from 50% to 100%. At 28 days, the strength decreased from 4.85 MPa to 4.15 MPa. At 90 days, the strength decreased from 5.40 MPa to 4.70 MPa. Although the increasing replacement ratio still weakened the strength, the composite strengthening treatment partially mitigated this adverse effect.

The strength improvement in the CR specimens was more pronounced at high recycled coarse aggregate replacement ratios. Taking the 90-day strength as an example, when the replacement ratio was 50%, the average strength increased from 4.72 MPa for the NCR specimen to 5.40 MPa for the CR specimen, representing a 14.4% increase. When the replacement ratio reached 100%, the average strength increased from 4.05 MPa to 4.70 MPa after composite strengthening, representing a 16.0% increase. This comparison indicates that the 5% NaOH + 10% PVA composite treatment improved the average unconfined compressive strength of high-content recycled aggregate cement-stabilized materials and helped compensate for the strength loss caused by the high replacement of natural coarse aggregate with recycled coarse aggregate.

In addition, the strength development coefficient, defined as the ratio of 90-day strength to 7-day strength, was used as an additional indicator to evaluate the long-term strength development of the specimens. For the CR specimen with 100% replacement of natural coarse aggregate by recycled coarse aggregate, the strength development coefficient was 1.65, close to the natural-aggregate reference value of 1.70. This result suggests that the composite-strengthened recycled aggregate cement-stabilized material maintained relatively stable long-term strength development even when natural coarse aggregate was fully replaced by recycled coarse aggregate.

The improvement in the CR specimens can be attributed to the combined effects of NaOH treatment and PVA modification. NaOH treatment may remove or weaken part of the loosely attached old mortar, while PVA modification may fill surface pores and microcracks and form a thin polymer film on the aggregate surface. These effects improve aggregate surface integrity and aggregate–matrix bonding, thereby enhancing stress transfer under compression. Overall, although strength decreased with increasing RCA replacement ratio, the CR specimens maintained higher average strength than the NCR specimens, indicating that the 5% NaOH + 10% PVA treatment improved the engineering applicability of high-content recycled aggregate cement-stabilized materials.

### 4.2. Splitting Tensile Strength

The splitting tensile strength was tested to evaluate the tensile resistance of the high-content recycled aggregate cement-stabilized materials. The specimen groups, RCA replacement ratios, and aggregate treatment conditions were the same as those described in Table 17. For each mixture, three replicate cylindrical specimens with dimensions of Ø100 mm × 100 mm were cured for 90 days and tested, and the average value was used as the representative splitting tensile strength.

The splitting tensile strength test was conducted after 90 days of curing using a YAW-3000 microcomputer-controlled automatic compression testing machine. During the test, each cylindrical specimen was placed horizontally between the loading platens, and the load was applied along the vertical diametral plane of the specimen until failure. The specimen axis was carefully aligned with the loading strip to reduce eccentric loading. The splitting tensile strength was calculated according to Equation (4):

(4) $$f_{t}=\frac{2P}{\pi DL}$$

where *f_t_* is the splitting tensile strength, (MPa); *P* is the peak failure load, (N); *D* is the specimen diameter, (mm); and *L* is the specimen height, (mm).

**Figure 10:**
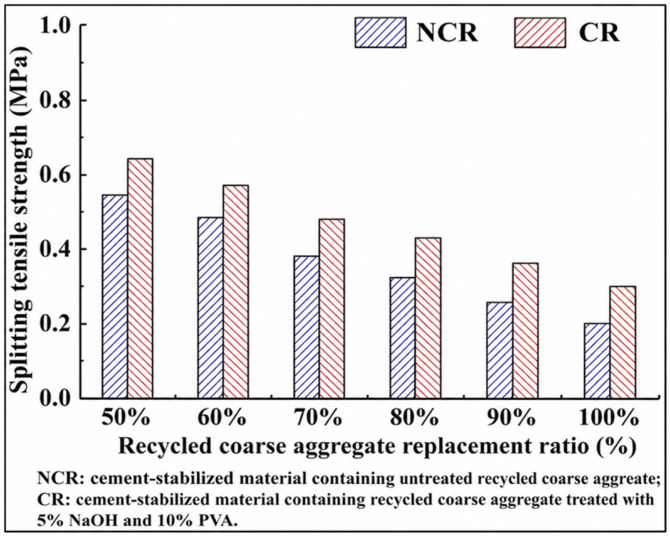
Average splitting tensile strength of high-content recycled aggregate cement-stabilized materials with different recycled coarse aggregate replacement ratios. NCR denotes mixtures prepared with particle-shaped recycled coarse aggregate without NaOH–PVA composite treatment, whereas CR denotes mixtures prepared with recycled coarse aggregate sequentially treated with 5% NaOH and 10% PVA.

The average splitting tensile strength results are shown in Figure 10.

As shown in Figure 10, the average splitting tensile strength of the NCR specimens decreased continuously as the recycled coarse aggregate replacement ratio increased. When the replacement ratio increased from 50% to 100%, the average splitting tensile strength decreased from 0.54 MPa to 0.20 MPa, corresponding to a reduction of 63.0%. This result indicates that increasing the content of particle-shaped recycled coarse aggregate without NaOH–PVA composite treatment had a pronounced adverse influence on the tensile resistance of high-content recycled aggregate cement-stabilized materials.

For the CR specimens, the average splitting tensile strength also decreased with increasing recycled coarse aggregate replacement ratio. The value decreased from 0.64 MPa at a 50% replacement ratio to 0.30 MPa at a 100% replacement ratio. However, at the same replacement ratio, the CR specimens consistently showed higher average splitting tensile strength than the corresponding NCR specimens. Compared with the NCR specimens, the average splitting tensile strength of the CR specimens increased by 18.5%, 20.8%, 26.3%, 31.3%, 38.5%, and 50.0% at replacement ratios of 50%, 60%, 70%, 80%, 90%, and 100%, respectively.

The improvement became more evident at higher recycled coarse aggregate replacement ratios. When the replacement ratio was 50%, the average splitting tensile strength increased from 0.54 MPa for the NCR specimen to 0.64 MPa for the CR specimen after composite strengthening. When the replacement ratio reached 100%, the average splitting tensile strength increased from 0.20 MPa to 0.30 MPa, corresponding to an increase of 50.0%. This comparison suggests that the 5% NaOH + 10% PVA composite treatment partially compensated for the tensile strength loss associated with the high incorporation of recycled coarse aggregate.

The decrease in splitting tensile strength with increasing recycled coarse aggregate content may be attributed to the inherent defects that remained in the particle-shaped recycled coarse aggregates. Compared with natural aggregates, recycled coarse aggregates generally contain residual old mortar, surface pores, and microcracks. These defects increase the heterogeneity of the cement-stabilized material and may weaken the interfacial region between recycled aggregates and the cementitious matrix. Since splitting tensile failure is highly sensitive to interfacial bonding and crack propagation, the deterioration of aggregate quality and interfacial bonding can reduce the tensile resistance of the material.

The improvement in the CR specimens can be associated with enhanced aggregate surface integrity and interfacial bonding after NaOH–PVA treatment. NaOH treatment may remove part of the weakly adhered old mortar, while PVA modification may fill surface pores and microcracks and form a thin film on the aggregate surface. These effects can improve the bonding condition between RCA and the cementitious matrix, delaying crack initiation and propagation under splitting tensile loading. Therefore, although splitting tensile strength decreased with increasing RCA replacement ratio, the CR specimens retained higher average tensile strength than the NCR specimens, particularly at high replacement ratios.

### 4.3. Compressive Rebound Modulus

The compressive rebound modulus was investigated to evaluate the stiffness and deformation resistance of high-content recycled aggregate cement-stabilized materials. A cement dosage of 5% by mass of the total dry aggregate was adopted as the benchmark cement content. In total, 13 groups of specimens were prepared, including one basic group without recycled coarse aggregate, denoted as BG, six groups containing recycled coarse aggregate without composite strengthening, denoted as NCR, and six groups containing recycled coarse aggregate strengthened by the optimized 5% NaOH + 10% PVA composite treatment, denoted as CR. The recycled coarse aggregate replacement ratios of the NCR and CR groups were 50%, 60%, 70%, 80%, 90%, and 100%.

The mixture compositions and controlled preparation parameters of the BG, NCR, and CR specimens are summarized in Table 17. The BG specimen was prepared with natural coarse aggregate and used as the reference group. The NCR specimens contained particle-shaped recycled coarse aggregate without NaOH–PVA composite treatment, whereas the CR specimens contained recycled coarse aggregate sequentially treated with 5% NaOH and 10% PVA. After curing at 20 ± 2 °C and a relative humidity of at least 95%, three replicate specimens were tested for each mixture and curing age. The arithmetic mean of the available measurements was used as the representative compressive rebound modulus.

The compressive rebound modulus was determined by the top-surface method specified in T 0808 of JTG E51-2009 [56]. The test was conducted using a YAW-3000 microcomputer-controlled automatic compression testing machine. Before loading, each specimen was centered on the loading platen, and the contact condition between the specimen and the loading surface was checked to reduce the influence of eccentric loading. During testing, two displacement transducers were symmetrically mounted on the loading platen along a diameter to record the axial deformation. Two loading–unloading preloading cycles were first applied at half of the predetermined maximum load. The maximum load, not exceeding 60–70% of the unconfined compressive strength determined from companion specimens, was divided into five to six equal steps.

At each loading step, the load was applied at a constant rate of 2.5 mm/min and maintained for 1 min before the loading reading was recorded. After unloading, the unloading reading was recorded following 0.5 min of elastic recovery. The recoverable deformation at each step was calculated as the difference between the loading and unloading readings. The unit pressure–recoverable deformation curve was then plotted, and its origin was corrected according to the standard procedure. Based on the corrected unit pressure–recoverable deformation relationship, the compressive rebound modulus was calculated using Equation (5):

(5) $$E_{c}=\frac{ph}{l}$$

where $E_{c}$ is the compressive rebound modulus (MPa); p is the calculated unit pressure on the loading platen (MPa); h is the specimen height (mm); and l is the recoverable deformation corresponding to *p* (mm). Considering the intended application of the material as a pavement base or subgrade material, the unit pressure *p* used for modulus calculation was selected within the range of 0.5–0.7 MPa in this study.

**Figure 11:**
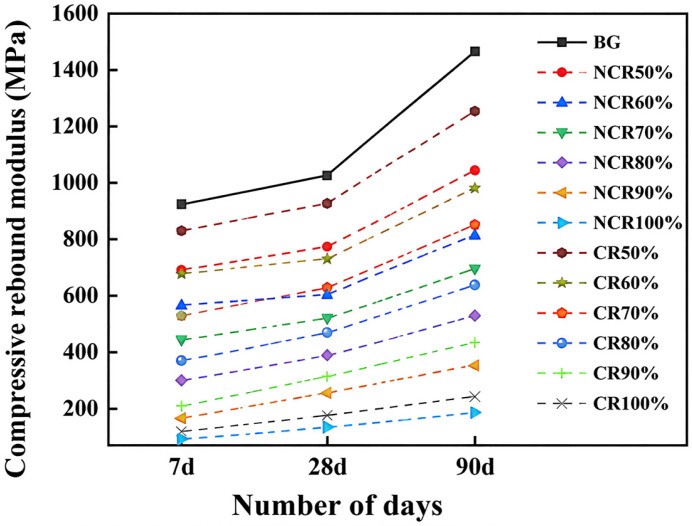
Average compressive rebound modulus of cement-stabilized material specimens prepared with different coarse aggregate conditions at recycled coarse aggregate replacement ratios of 50–100% and curing ages of 7, 28, and 90 days. BG denotes the benchmark cement-stabilized material prepared with natural coarse aggregate; NCR denotes cement-stabilized material prepared with particle-shaped recycled coarse aggregate without NaOH–PVA composite treatment; CR denotes cement-stabilized material prepared with recycled coarse aggregate sequentially treated with 5% NaOH and 10% PVA.

Measurements affected by abnormal specimen failure, eccentric loading, or obvious testing deviation would be considered invalid in accordance with the test procedure. The reported results in Figure 11 represent the arithmetic means of the available measurements at each replacement ratio and curing age.

As shown in Figure 11, the compressive rebound modulus of all specimens generally increased with curing age under standard curing conditions. For the BG specimen, the modulus increased from 920 MPa at 7 days to 1025 MPa at 28 days and further increased to 1480 MPa at 90 days. Similar age-dependent increases were also observed in the recycled aggregate specimens. This trend indicates that the continued hydration of cementitious materials and the gradual densification of the matrix contributed to the development of stiffness with increasing curing age.

At the same curing age, the compressive rebound modulus decreased as the recycled coarse aggregate replacement ratio increased. For the NCR specimens, as the replacement ratio increased from 50% to 100%, the average modulus values showed a continuous downward trend. Compared with the BG specimen, the modulus of the specimen with 100% recycled coarse aggregate was much lower. Specifically, the modulus values decreased from 920 MPa, 1025 MPa, and 1480 MPa for the BG specimen to 120 MPa, 168 MPa, and 225 MPa for the NCR-100 specimen at 7, 28, and 90 days, respectively. The corresponding reductions were 86.9%, 83.6%, and 84.8%, indicating that a high recycled coarse aggregate content had a pronounced adverse effect on the deformation resistance of cement-stabilized materials.

For the NCR specimen with a 70% recycled coarse aggregate replacement ratio, the compressive rebound modulus values at 7, 28, and 90 days were 450 MPa, 535 MPa, and 720 MPa, respectively. Compared with the BG specimen, these values decreased by approximately 51.1%, 47.8%, and 51.4%, respectively. This result shows that even at a moderate-to-high recycled coarse aggregate replacement ratio, the stiffness of the cement-stabilized material was clearly lower than that of the reference specimen without recycled coarse aggregate.

After composite strengthening with 5% NaOH + 10% PVA, the compressive rebound modulus of the recycled aggregate specimens increased to varying degrees. For the CR specimen with a 70% replacement ratio, the modulus values at 7, 28, and 90 days increased to 540 MPa, 642 MPa, and 864 MPa, respectively. Compared with the corresponding NCR specimen, the increases were approximately 20.0% at all three curing ages. This comparison indicates that the composite strengthening treatment improved the stiffness and deformation resistance of the recycled aggregate cement-stabilized material.

The reduction in compressive rebound modulus with increasing recycled coarse aggregate replacement ratio may be attributed to the physical defects of recycled aggregates. Compared with natural aggregates, recycled coarse aggregates generally contain residual old mortar, internal microcracks, and a more porous surface structure. These defects reduce the intrinsic stiffness of the aggregate phase and increase the heterogeneity of the material. In addition, the old mortar attached to recycled aggregates may form relatively weak interfacial regions between the recycled aggregates and the new cementitious matrix. Under compressive loading, these weak regions are more prone to microcrack initiation and deformation accumulation, resulting in a lower rebound modulus.

The decrease in modulus was more obvious at high recycled coarse aggregate replacement ratios because the proportion of recycled aggregate particles and attached old mortar increased in the load-bearing skeleton. When the replacement ratio reached 90% or 100%, the skeleton of the cement-stabilized material was mainly composed of recycled aggregates. In this case, the higher water absorption, lower crushing resistance, and greater porosity of recycled aggregates had a stronger influence on the overall stiffness. Therefore, the compressive rebound modulus decreased more markedly as the recycled coarse aggregate content increased.

The increase in compressive rebound modulus after NaOH–PVA treatment can be attributed to improved RCA surface quality and aggregate–matrix interfacial bonding. NaOH treatment may weaken loosely attached old mortar, while PVA modification may seal surface pores and microcracks. These changes reduce local defects around RCA particles and promote more uniform stress transfer between the aggregate skeleton and cementitious matrix. Nevertheless, when the RCA replacement ratio was very high, the lower stiffness, higher porosity, and weaker interfaces associated with RCA still dominated the deformation response. Overall, the modulus results are consistent with the strength results in Section 4.1 and Section 4.2: increasing RCA replacement reduced stiffness, whereas composite treatment partially mitigated this reduction.

### 4.4. Freeze–Thaw Cycle Test

Considering the severe cold climatic conditions in Xinjiang, an indoor freeze–thaw cycle test was conducted to evaluate the frost resistance of high-content recycled aggregate cement-stabilized materials. According to the *Technical Code for Recycling of Construction and Demolition Waste* (GB/T 50743-2012), materials intended for use in severely cold regions should be evaluated after at least 50 freeze–thaw cycles [57]. Therefore, 50 freeze–thaw cycles were adopted in this study, corresponding to the F50 evaluation condition.

The freeze–thaw test was conducted using the same cement-stabilized material system and controlled preparation parameters described in Table 17. Seven RCA replacement ratios, namely 0%, 50%, 60%, 70%, 80%, 90%, and 100%, were investigated. The 0% replacement ratio served as the natural aggregate reference group, while the RCA-containing specimens included particle-shaped RCA without NaOH–PVA composite treatment (NCR) and composite-modified RCA treated sequentially with 5% NaOH and 10% PVA (CR).

Before freeze–thaw cycling, the specimens were immersed in water to reach a saturated surface-dry condition. Freeze–thaw cycling was performed using a Changsha Yaxing YXDR-1 rapid freeze–thaw testing machine (Changsha, China), with freezing and thawing temperatures of −20 ± 2 °C and 20 ± 2 °C, respectively. The holding time at each constant-temperature stage was not less than 4 h. For each mixture, three parallel specimens were tested. Mass loss was calculated from the specimen masses before and after freeze–thaw cycling, and residual compressive strength was determined after 50 cycles using a YAW-3000 compression testing machine. The frost-resistance coefficient was calculated as the ratio of the compressive strength after 50 freeze–thaw cycles to the corresponding compressive strength before freeze–thaw cycling. The mass loss rate and frost-resistance coefficient were used to evaluate freeze–thaw resistance. According to the relevant durability requirements, the mass loss rate should not exceed 5%, and the frost-resistance coefficient should not be lower than 0.75 [57,58].

**Figure 12:**
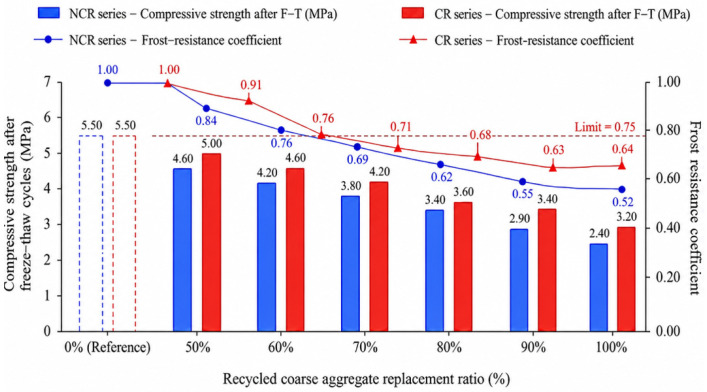
Residual compressive strength and frost-resistance coefficient of high-content recycled aggregate cement-stabilized materials after 50 freeze–thaw cycles.

The mean freeze–thaw test results are summarized in Table 18, while Figure 12 presents the variations in residual compressive strength and frost-resistance coefficient with RCA replacement ratio.

As shown in Figure 12 and Table 18, the residual compressive strength generally decreased with increasing RCA replacement ratio in both series after 50 freeze–thaw cycles. For the NCR series, the residual compressive strength decreased from 4.60 MPa at 50% RCA replacement to 2.40 MPa at 100% RCA replacement, corresponding to a reduction of 47.8%. For the CR series, the residual compressive strength decreased from 5.00 MPa to 3.20 MPa over the same replacement range, corresponding to a reduction of 36.0%. Compared with the NCR series, the residual compressive strengths of the CR series were higher by 8.7%, 9.5%, 10.5%, 5.9%, 17.2%, and 33.3% at RCA replacement ratios of 50%, 60%, 70%, 80%, 90%, and 100%, respectively. These results indicate that the composite modification improved residual strength retention after freeze–thaw cycling.

The frost-resistance coefficient showed a similar overall decreasing trend with increasing RCA replacement ratio. For the NCR series, the coefficient decreased from 0.84 at 50% RCA replacement to 0.52 at 100% RCA replacement. The coefficients at 50% and 60% replacement were 0.84 and 0.76, respectively, meeting the specified limit of 0.75. However, the coefficients at 70%, 80%, 90%, and 100% replacement were 0.69, 0.62, 0.55, and 0.52, respectively, indicating that these mixtures did not satisfy the F50 frost-resistance requirement.

For the CR series, the frost-resistance coefficients were higher than those of the corresponding NCR series at RCA replacement ratios of 50%, 70%, 80%, 90%, and 100%. At the 60% replacement ratio, however, both series showed the same coefficient of 0.76. The CR series met the F50 requirement at 50% and 60% RCA replacement, with coefficients of 0.91 and 0.76, respectively, whereas the coefficients at 70–100% replacement ranged from 0.71 to 0.64 and remained below 0.75. Therefore, the improvement in frost resistance should be interpreted from the overall trend rather than as superiority at every individual replacement ratio.

Table 18 further presents the mass loss results and corresponding standard deviations. The mass loss values of all mixtures remained below 5%, ranging from 0.55 ± 0.05% to 3.35 ± 0.18%, thereby satisfying the mass loss criterion. Nevertheless, mass loss generally increased with increasing RCA replacement ratio in both series. At the same RCA replacement ratio, the CR series showed lower mass loss than the NCR series. For example, at 100% RCA replacement, the mass loss decreased from 3.35 ± 0.18% for the NCR series to 2.55 ± 0.14% for the CR series. The relatively small standard deviations indicate acceptable consistency among the parallel specimens. Together with the residual compressive strength and frost-resistance coefficient results, these findings suggest that the composite modification reduced freeze–thaw-induced surface scaling and internal deterioration to some extent.

Freeze–thaw deterioration is mainly related to the porous old mortar attached to RCA and damage caused by pore-water phase changes. During freezing, water in pores and interfacial regions expands and generates internal stress; during thawing, repeated water migration and redistribution further promote microcrack development [31,33,58]. As the RCA replacement ratio increases, the amount of attached old mortar and the proportion of weak interfacial regions also increase, resulting in greater accumulated freeze–thaw damage.

The better overall performance of the CR series may be associated with the combined effects of NaOH treatment and PVA modification. NaOH treatment may weaken or remove part of the loosely attached old mortar, while PVA modification may fill surface pores and microcracks and form a polymer film on the aggregate surface. These effects can reduce water ingress and improve the compactness of the aggregate–matrix interface, thereby delaying frost-induced microcracking and reducing mass loss. However, when the RCA replacement ratio exceeded 60%, the frost-resistance coefficients of both the NCR and CR series were lower than 0.75, indicating that aggregate surface modification alone was insufficient to fully offset the accumulated defects caused by high RCA contents.

In summary, under the F50 freeze–thaw condition, all mixtures satisfied the mass loss requirement, whereas the frost-resistance coefficient decreased with increasing RCA replacement ratio. Both the NCR and CR series met the F50 frost-resistance requirement when the RCA replacement ratio did not exceed 60%. For engineering applications in severely cold regions of Xinjiang, an RCA replacement ratio not exceeding 60% is therefore recommended for the investigated cement-stabilized material system. At higher RCA replacement ratios, additional measures, such as matrix densification, secondary carbonation, silica fume incorporation, or air entrainment, may be required to further improve long-term freeze–thaw durability [59,60].

### 4.5. Drying Shrinkage Performance

Drying shrinkage is an important factor affecting the volume stability and long-term performance of cement-stabilized materials, particularly when they are used in pavement base or subgrade engineering in arid and cold regions [61]. In this study, the drying shrinkage behavior of high-content recycled aggregate cement-stabilized materials was investigated by considering the effects of recycled coarse aggregate replacement ratio and composite aggregate strengthening. The objective was to clarify the influence of recycled coarse aggregate incorporation and 5% NaOH + 10% PVA composite treatment on shrinkage development and volume stability.

The drying shrinkage test was conducted using the same cement-stabilized material system as that described in Section 4.1, Section 4.2, Section 4.3 and Section 4.4. A two-factor experimental design was adopted. The recycled coarse aggregate replacement ratio was used as the primary variable, with seven levels: 0%, 50%, 60%, 70%, 80%, 90%, and 100%. The 0% replacement ratio was used as the natural aggregate reference group. For the recycled aggregate specimens, two aggregate treatment conditions were considered: particle-shaped recycled coarse aggregate without NaOH–PVA composite treatment and composite-strengthened recycled coarse aggregate treated with 5% NaOH and 10% PVA. The main design parameters and controlled preparation conditions were consistent with those summarized in Table 17.

The mixtures were prepared using an HJW-30 single-horizontal-shaft forced concrete mixer to ensure uniform distribution of cementitious materials, aggregates, and mixing water. Before mixing, the recycled coarse aggregates were adjusted to a saturated surface-dry condition as far as possible to reduce the influence of aggregate water absorption on the effective water content of the mixture. The cement dosage was fixed at 5% by mass of the total dry aggregate, consistent with the mechanical performance and freeze–thaw tests in this section.

Prismatic specimens with dimensions of 100 mm × 100 mm × 515 mm were prepared, with three replicate specimens for each group. After molding, the specimens were covered to reduce rapid moisture loss and then cured in an SHBY-40B standard constant-temperature and constant-humidity curing chamber. The drying shrinkage test was conducted using an HSP-540 concrete shrinkage–expansion tester (Hebei Xinsheng Testing Instrument Co., Ltd., Cangzhou, China) in accordance with the Standard *Test Methods for Long-Term Performance and Durability of Ordinary Concrete* (GB/T 50082-2009) [58].

After standard curing for 3 days, the specimens were transferred to a constant-temperature and constant-humidity environment for drying shrinkage measurement. The length change in each specimen was recorded using a dial gauge measurement system at the specified ages. The drying shrinkage strain was calculated according to Equation (6):

(6) $$\varepsilon_{t}=\frac{L_{0}-L_{t}}{L}\times 10^{6}(\mu \varepsilon )$$

where $\varepsilon_{t}$ is the drying shrinkage strain at age t, (με); *L*_0_ is the initial length reading, (mm); *L*_t_ is the length reading at age t, (mm); and *L* is the effective gauge length of the specimen, (mm).

**Figure 13:**
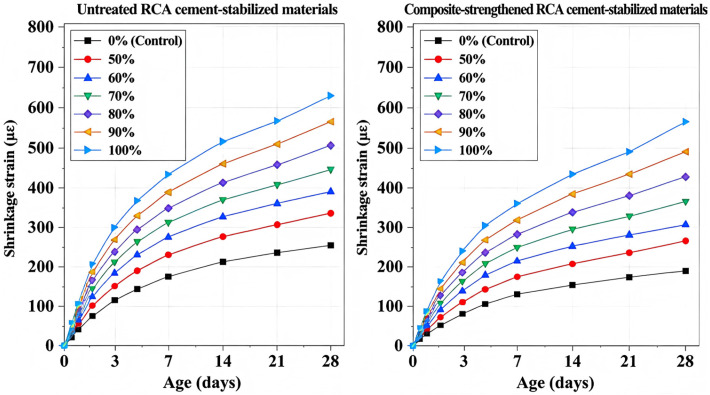
Drying shrinkage strain of NCR and CR cement-stabilized materials with different RCA replacement ratios.

In this study, the effective gauge length was determined as the specimen length minus twice the probe insertion depth. The average drying shrinkage strain results are shown in Figure 13.

As shown in Figure 13, the drying shrinkage strain increased with increasing recycled coarse aggregate replacement ratio. The 28-day drying shrinkage strain of the reference group with 0% recycled coarse aggregate was 264 με. For the specimens containing untreated recycled coarse aggregate, the 28-day shrinkage strain increased from 351 με at a 50% replacement ratio to 646 με at a 100% replacement ratio. On average, each 10% increase in recycled coarse aggregate content resulted in an approximately 49.3 με increase in the 28-day shrinkage strain, indicating a clear increasing trend between shrinkage deformation and recycled coarse aggregate replacement ratio.

After composite strengthening with 5% NaOH + 10% PVA, the drying shrinkage strain of each recycled aggregate specimen group was reduced to varying degrees. For the 50% replacement group, the 28-day shrinkage strain decreased from 351 με to 290 με, corresponding to a reduction of 17.4%. For the 100% replacement group, the shrinkage strain decreased from 646 με to 592 με, corresponding to a reduction of 8.4%. These results indicate that the composite strengthening treatment reduced drying shrinkage deformation, although the reduction became less pronounced as the recycled coarse aggregate replacement ratio increased.

From the perspective of time-dependent shrinkage development, the shrinkage strain of all groups increased rapidly during the early drying period and then gradually stabilized. The increase was more pronounced from 3 to 14 days, while the growth rate decreased after 14 days. For example, the reference group reached a shrinkage strain of 198 με at 14 days, with an average growth rate of 18.5 με/day during the early stage. From 14 to 28 days, the strain increased by only 66 με, corresponding to an average growth rate of 5.3 με/day. This indicates that drying shrinkage mainly developed at early ages, when moisture evaporation from the cementitious matrix and internal capillary water migration occurred relatively rapidly.

For the untreated group with a 70% recycled coarse aggregate replacement ratio, a relatively rapid increase in shrinkage was observed between 3 and 7 days. The shrinkage strain reached 300 με at 7 days, representing an increase of 74.4% compared with that at 3 days. In contrast, the corresponding composite-strengthened group showed a 65.8% increase over the same period. This comparison suggests that the 5% NaOH + 10% PVA treatment may reduce early-age shrinkage deformation, particularly in specimens with relatively high recycled coarse aggregate contents.

The increase in drying shrinkage with recycled coarse aggregate content may be attributed to the physical characteristics of recycled coarse aggregates. Compared with natural aggregates, recycled coarse aggregates generally contain residual old mortar, surface pores, and microcracks. These defects increase water absorption and provide more channels for moisture migration during drying. As the recycled coarse aggregate replacement ratio increases, the amount of attached old mortar and the extent of porous interfacial regions in the material also increase, leading to greater internal moisture loss and higher shrinkage deformation.

In addition, recycled coarse aggregates usually have a lower elastic modulus and a weaker restraining effect than natural aggregates. In cement-stabilized materials, coarse aggregates act as a restraining skeleton that limits shrinkage deformation of the cementitious matrix. However, when a large amount of recycled coarse aggregate is incorporated, the attached old mortar and relatively weak aggregate–matrix interfaces may reduce this restraining effect [62,63]. As a result, shrinkage deformation of the cementitious matrix is more readily transmitted to the whole specimen, leading to higher drying shrinkage strain.

Interfacial characteristics are another important factor influencing shrinkage behavior. Cement-stabilized materials containing recycled aggregates involve multiple interfaces, including those between the original aggregate and old mortar, old mortar and new cementitious matrix, and recycled aggregate and new matrix. These interfacial regions are generally more porous and weaker than those in materials prepared with natural aggregates. During drying, moisture loss and capillary tension may concentrate in these weak regions, promoting microcrack initiation and accelerating shrinkage development. This mechanism is consistent with the mechanical and durability deterioration discussed in Section 4.1, Section 4.2, Section 4.3 and Section 4.4.

The reduction in shrinkage after composite strengthening can be attributed to improved RCA surface quality and reduced moisture migration. NaOH treatment may remove or modify part of the weak residual mortar, while PVA modification may fill surface pores and microcracks and form a thin polymer film after drying. These effects can improve the aggregate–matrix interface and reduce connected moisture migration channels, leading to lower drying shrinkage strain in the CR specimens [27,28,52].

However, the strengthening effect decreased at higher RCA replacement ratios. When the replacement ratio increased to 100%, the shrinkage reduction was only 8.4%, compared with 17.4% at 50% replacement. This indicates that, when RCA dominates the aggregate skeleton, the accumulated effects of attached old mortar, high porosity, and multiple weak interfaces still control shrinkage development. Therefore, for pavement base or subgrade applications requiring good volume stability, composite strengthening should be combined with appropriate control of the RCA replacement ratio and, if necessary, additional measures such as matrix densification, optimized gradation, shrinkage-reducing admixtures, or improved curing conditions.

## 5. Conclusions

This study investigated integrated strengthening methods for recycled coarse aggregates derived from construction and demolition waste in Aksu, Xinjiang. The effects of these methods on recycled aggregate concrete and high-content recycled aggregate cement-stabilized materials were evaluated. Particle shaping, alkaline treatment, PVA modification, and cementitious matrix optimization were adopted to improve the quality of recycled coarse aggregates and the mechanical and durability performance of the prepared materials. Based on the experimental results, the following conclusions can be drawn.

1. The untreated recycled coarse aggregate with particle sizes of 4.75–31.5 mm contained attached old mortar, surface pores, and microcracks, resulting in relatively poor physical properties. Its water absorption, crushing index, and apparent density were 6.8%, 14.4%, and 2588 kg/m^3^, respectively. According to GB/T 25177-2010, the untreated recycled coarse aggregate was classified as Class III recycled coarse aggregate. This indicates that its direct use may be limited in recycled aggregate concrete or cement-stabilized materials with relatively high mechanical or durability requirements.
2. Particle shaping, NaOH treatment, and PVA modification improved the physical properties of recycled coarse aggregate. Particle shaping reduced the water absorption and crushing index to 5.6% and 12.6%, respectively. Among the alkaline treatments, 5% NaOH showed the most favorable improvement, reducing the water absorption to 3.9% and the crushing index to 9.3%. After further modification with 10% PVA, the NHP-5 group reached an apparent density of 2779 kg/m^3^, a water absorption of 3.3%, and a crushing index of 8.3%, satisfying the Class II recycled coarse aggregate requirement. These improvements may be attributed to the removal or modification of weak residual mortar, partial filling of surface pores and microcracks, and improved surface compactness of the recycled aggregate.
3. The combined scheme of 5% NaOH + 10% PVA aggregate treatment and cementitious matrix optimization with 10% silica fume and 0.2% polycarboxylate-based water-reducing agent improved the compressive strength of recycled aggregate concrete. Under the tested mix conditions, the 28-day compressive strength reached 27.6 MPa, corresponding to 97.5% of that of natural aggregate concrete. This indicates that properly strengthened recycled aggregate concrete can approach the compressive strength level of natural aggregate concrete under the present experimental conditions. SEM observations qualitatively suggested that the improvement was associated with a denser interfacial transition zone, fewer visible pores and microcracks, and a more continuous distribution of hydration products. However, these microstructural interpretations should be regarded as qualitative evidence supporting the macroscopic test results.
4. For high-content recycled aggregate cement-stabilized materials with recycled coarse aggregate replacement ratios of 50–100%, the specimens prepared with composite-strengthened recycled coarse aggregate generally exhibited better mechanical performance than those prepared with untreated recycled coarse aggregate. At a 100% replacement ratio, the 90-day unconfined compressive strength and splitting tensile strength reached 4.70 MPa and 0.30 MPa, representing increases of 16.0% and 50.0%, respectively, compared with the corresponding unmodified group. The compressive rebound modulus was also improved after aggregate modification. These results indicate that the 5% NaOH + 10% PVA treatment can partially mitigate the reductions in strength and stiffness caused by high recycled coarse aggregate contents, mainly by improving aggregate quality and enhancing the aggregate–matrix interfacial condition.
5. In high-content recycled aggregate cement-stabilized materials, increasing the recycled coarse aggregate replacement ratio adversely affected frost resistance and drying shrinkage performance. After 50 freeze–thaw cycles, the frost resistance coefficient decreased as the recycled coarse aggregate replacement ratio increased. Although aggregate modification improved the residual compressive strength and frost resistance coefficient, the F50 requirement was satisfied only when the replacement ratio did not exceed 60% under the present test conditions. In terms of drying shrinkage, the modified specimens showed reductions in 28-day shrinkage strain of 17.4% and 8.4% at replacement ratios of 50% and 100%, respectively. Therefore, for engineering applications in severely cold regions such as Xinjiang, the recycled coarse aggregate replacement ratio is recommended to be controlled at no more than 60% when the current material system and strengthening process are adopted.
6. Overall, the proposed strengthening system improved the physical quality of recycled coarse aggregates and enhanced the mechanical and durability performance of recycled aggregate concrete and high-content recycled aggregate cement-stabilized materials. However, this study has several limitations. The recycled aggregates were obtained from a single regional source, and the durability evaluation was limited to laboratory freeze–thaw and drying shrinkage tests. In addition, because no formal statistical analysis was performed and some experimental records retained only mean values, the variability and statistical significance of the observed differences could not be fully evaluated. Moreover, this study did not include a PVA-only aggregate treatment group or separate silica-fume-only and water-reducing-agent-only matrix optimization groups; therefore, the individual contribution of each component could not be fully separated. The observed performance improvement should therefore be interpreted as the combined effect of the adopted strengthening measures rather than conclusive evidence of a quantitatively verified synergistic mechanism. Future work should adopt a factorial experimental design, retain complete individual test data, and incorporate more comprehensive statistical evaluations, including standard deviations, coefficients of variation, confidence intervals, and error bars. Long-term field performance, chloride penetration, carbonation resistance, sulfate attack, coupled environmental actions, and the cost, environmental impact, construction efficiency, and large-scale applicability of the strengthening process should also be further investigated.

## Figures and Tables

**Table 1:** Chemical composition of recycled coarse aggregate.

Chemical Composition	CaO	SiO_2_	Al_2_O_3_	Fe_2_O_3_	SO_3_	MgO	Na_2_O	K_2_O	BaO
Content (wt.%)	75.34	15.58	6.25	1.38	0.26	0.45	0.25	0.31	0.18

**Table 2:** Classification criteria and application scope of recycled coarse aggregates.

Class	Water Absorption by Mass (%)	Crushing Index (%)	Apparent Density (kg/m^3^)	Application Scope
Class I	<3.0	<12	>2450	Suitable for concrete of all strength grades
Class II	<5.0	<20	>2350	Suitable for concrete with strength grades of C40 and below
Class III	<8.0	< 30	>2250	Suitable for concrete with strength grades of C25 and below, but not recommended for concrete with frost resistance requirements

**Table 3:** Water absorption of natural and recycled coarse aggregates.

Aggregate Type	Natural Coarse Aggregate	Recycled Coarse Aggregate
Water absorption (%)	1.38	6.82

**Table 4:** Crushing index of natural and recycled coarse aggregates.

Aggregate Type	Natural Coarse Aggregate	Recycled Coarse Aggregate
Crushing Index (%)	5.2	14.4

**Table 5:** Apparent density of natural and recycled coarse aggregates.

Aggregate Type	Natural Coarse Aggregate	Recycled Coarse Aggregate
Apparent density (kg/m^3^)	2787	2588

**Table 6:** Water absorption and crushing index of different coarse aggregates.

Aggregate Type	Water Absorption (%)	Crushing Index (%)
Recycled coarse aggregate	6.8	14.4
Particle-shaped recycled coarse aggregate	5.6	12.6
Natural pebble	1.38	5.2

**Table 7:** Bulk density and apparent density of recycled aggregates before and after particle shaping.

Aggregate Type	Bulk Density (kg/m^3^)	Apparent Density (kg/m^3^)
Recycled aggregate	1326	2588
Particle-shaped recycled aggregate	1458	2678
Natural aggregate, pebble	1606	2787

**Table 8:** Mix proportions of concrete prepared with different aggregate types.

Concrete Type	Nominal Water-to-Cement Ratio	Water/kg/m^3^	Cement/kg/m^3^	Medium Sand/kg/m^3^	Coarse Aggregate/kg/m^3^
Recycled coarse aggregate concrete, RCA	0.41	175	427	525	1286
Particle-shaped recycled aggregate concrete, TRCA	0.41	175	427	525	1286
Natural pebble aggregate concrete, NA	0.41	175	427	525	1286

**Table 9:** Compressive strength of concrete prepared with different aggregate types.

Concrete Type	7-Day Compressive Strength (MPa)	28-Day Compressive Strength (MPa)
Recycled coarse aggregate concrete (RCA)	10.3	14.3
Particle-shaped recycled aggregate concrete (TRCA)	12.3	17.6
Natural pebble aggregate concrete (NA)	21.3	28.3

**Table 10:** Mix proportions of concrete prepared with alkali-treated recycled coarse aggregates.

Number	Nominal Water-to-Cement Ratio	Water (kg/m^3^)	Cement (kg/m^3^)	Medium Sand (kg/m^3^)	Coarse Aggregate (kg/m^3^)
Z	0.41	175	427	525	1286
N-2	0.41	175	427	525	1286
N-3	0.41	175	427	525	1286
N-4	0.41	175	427	525	1286
N-5	0.41	175	427	525	1286
N-6	0.41	175	427	525	1286
NH-2	0.41	175	427	525	1286
NH-3	0.41	175	427	525	1286
NH-4	0.41	175	427	525	1286
NH-5	0.41	175	427	525	1286
NH-6	0.41	175	427	525	1286
T	0.41	175	427	525	1286

**Table 11:** Compressive strength of concrete prepared with alkali-treated recycled coarse aggregates.

Number	7-Day Compressive Strength (MPa)	28-Day Compressive Strength (MPa)
Z	10.2	14.4
N-2	13.1	18.3
N-3	13.5	18.9
N-4	13.8	19.7
N-5	14.2	19.5
N-6	14.0	19.4
NH-2	13.8	19.9
NH-3	14.5	21.2
NH-4	16.3	22.4
NH-5	16.8	24.1
NH-6	16.2	23.6
T	21.3	28.3

**Table 12:** Bulk density, apparent density, water absorption, and crushing index of recycled coarse aggregates after alkaline solution–PVA composite treatment.

Aggregate Type	Bulk Density (kg/m^3^)	Apparent Density (kg/m^3^)	Water Absorption (%)	Crushing Index (%)
NP-4	1518	2705	4.2	10.6
NP-5	1521	2728	4.0	10.2
NHP-4	1552	2757	3.6	9.2
NHP-5	1564	2779	3.3	8.3
Untreated Recycled Aggregate	1326	2588	6.8	14.4
Natural pebble aggregate	1606	2787	1.38	5.2

**Table 13:** Mix proportions of concrete prepared with composite-modified recycled coarse aggregates.

Number	Nominal Water-to-Cement Ratio	Water (kg/m^3^)	Cement (kg/m^3^)	Medium Sand (kg/m^3^)	Coarse Aggregate (kg/m^3^)
NP-4	0.41	175	427	525	1286
NP-5	0.41	175	427	525	1286
NHP-4	0.41	175	427	525	1286
NHP-5	0.41	175	427	525	1286

**Table 14:** Compressive strength of concrete prepared with composite-modified recycled coarse aggregates.

Number	7-Day Compressive Strength (MPa)	28-Day Compressive Strength (MPa)
NP-4	14.4	20.8
NP-5	15.8	20.6
NHP-4	16.7	23.8
NHP-5	18.2	25.7

**Table 15:** Mix proportions of concrete with combined strengthening of recycled coarse aggregate and cementitious matrix.

Number	Nominal Water-to-Cement Ratio	Water (kg/m^3^)	Cement (kg/m^3^)	Silica Fume (kg/m^3^)	Water-Reducing Agent (kg/m^3^)	Medium Sand (kg/m^3^)	Coarse Aggregate (kg/m^3^)
NP-4	0.41	175	427	42.7	0.85	525	1286
NP-5	0.41	175	427	42.7	0.85	525	1286
NHP-4	0.41	175	427	42.7	0.85	525	1286
NHP-5	0.41	175	427	42.7	0.85	525	1286
T	0.41	175	427	—	—	525	1286

**Table 16:** Compressive strength of concrete with combined strengthening of recycled coarse aggregate and cementitious matrix.

Number	7-Day Compressive Strength (MPa)	28-Day Compressive Strength (MPa)
NP-4	15.2	24.5
NP-5	16.5	25.3
NHP-4	17.6	26.2
NHP-5	19.9	27.6
T	21.3	28.3

**Table 17:** Complete mixture compositions and controlled preparation parameters of high-content recycled aggregate cement-stabilized materials in Section 4.

Group	RCA Replacement Ratio (%)	Cement (g)	Water (g)	Fine Aggregate, Dry (g)	Natural Coarse Aggregate, Dry (g)	Recycled Coarse Aggregate, Dry (g)	Total Dry Aggregate (g)	Specimen Size
BG	0	84.1	101.0	504.9	1178.0	0	1682.9	Ø100 mm × 100 mm
NCR-50; CR-50	50	84.1	101.0	504.9	589.0	589.0	1682.9	Ø100 mm × 100 mm
NCR-60; CR-60	60	84.1	101.0	504.9	471.2	706.8	1682.9	Ø100 mm × 100 mm
NCR-70; CR-70	70	84.1	101.0	504.9	353.4	824.6	1682.9	Ø100 mm × 100 mm
NCR-80; CR-80	80	84.1	101.0	504.9	235.6	942.4	1682.9	Ø100 mm × 100 mm
NCR-90; CR-90	90	84.1	101.0	504.9	117.8	1060.2	1682.9	Ø100 mm × 100 mm
NCR-100; CR-100	100	84.1	101.0	504.9	0	1178.0	1682.9	Ø100 mm × 100 mm

Note: The cement dosage was 5% of the total dry aggregate mass, and the molding water content was 6.0%. The fine and coarse aggregate fractions accounted for 30% and 70% of the total dry aggregate mass, respectively. The recycled coarse aggregate replaced natural coarse aggregate by mass at the specified replacement ratios. All specimens were formed by static compaction at 2.5 MPa for 60 s, with a target compactness of not less than 98%, and were cured under standard conditions of 20 ± 2 °C and relative humidity ≥ 95%.

**Table 18:** Mean residual compressive strength, frost-resistance coefficient, and mass loss of high-content recycled aggregate cement-stabilized materials after 50 freeze–thaw cycles.

RCA Replacement Ratio (%)	Series	Compressive Strength After F–T Cycles (MPa)	Frost-Resistance Coefficient	Mass Loss After F–T Cycles (%)
0	Reference	5.5	1.00	0.55 ± 0.05
50	NCR	4.6	0.84	1.22 ± 0.07
50	CR	5.0	0.91	0.95 ± 0.06
60	NCR	4.2	0.76	1.55 ± 0.09
60	CR	4.6	0.76	1.28 ± 0.07
70	NCR	3.8	0.69	1.95 ± 0.11
70	CR	4.2	0.71	1.62 ± 0.09
80	NCR	3.4	0.62	2.38 ± 0.13
80	CR	3.6	0.68	2.12 ± 0.12
90	NCR	2.9	0.55	2.85 ± 0.15
90	CR	3.4	0.63	2.25 ± 0.13
100	NCR	2.4	0.52	3.35 ± 0.18
100	CR	3.2	0.64	2.55 ± 0.14

Note: Mass loss values are presented as mean ± standard deviation of three specimens. NCR: particle-shaped recycled coarse aggregate without NaOH–PVA composite treatment; CR: recycled coarse aggregate treated sequentially with 5% NaOH and 10% PVA.

## Data Availability

The original contributions presented in this study are included in the article. Further inquiries can be directed to the corresponding author.
